# Competency assessment of the medical interns and nurses and documenting prevailing practices to provide family planning services in teaching hospitals in three states of India

**DOI:** 10.1371/journal.pone.0211168

**Published:** 2019-11-06

**Authors:** Madhu Gupta, Madhur Verma, Kiranjit Kaur, Kirti Iyengar, Tarundeep Singh, Anju Singh

**Affiliations:** 1 Department of Community Medicine and School of Public Health, Postgraduate Institute of Medical Education and Research, Chandigarh, India; 2 United Nations Population Fund, New Delhi, India; 3 Department of Obstetrics and Gynaecology, Postgraduate Institute of Medical Education and Research, Chandigarh, India; Liverpool School of Tropical Medicine, UNITED KINGDOM

## Abstract

**Objectives:**

The objectives of the study were to assess the knowledge and skills of medical interns and nurses regarding family planning (FP) services, and document the prevailing FP practices in the teaching hospitals in India.

**Study design:**

A cross-sectional study was conducted in three states (Delhi, Rajasthan, and Maharashtra) of India, among randomly selected 163 participants, including medical interns (n = 81) and in-service nurses (n = 82), during 2017. The semi-structured, pre-tested interview schedule, was used to assess the knowledge and status of training received; and objective structured clinical examination (OSCE) based checklist was used to evaluate the skills.

**Results:**

About 60% of the interns and 48% of the nurses knew more than five contraceptives that could be offered to the clients. About 22% (11.1% interns and 33.3% nurses) respondents believed that contraceptives should not be given to a married woman coming alone, and 31.9% (17.3% interns and 46.3% nurses) respondents reported that it was illegal to provide contraceptives to unmarried people. Nearly 43.3% interns and 69.5% nurses refused to demonstrate intrauterine contraceptive device (IUCD) insertion in the dummy uterus as per OSCE, and among those who did, 12.3% interns and 18.3% nurses had failed. About 63% interns and 63.4% of nurses had observed IUCD insertion, and 12.3% interns and 17.1% had performed IUCD insertion, during their training.

**Conclusions:**

Knowledge and skills of interns and nurses regarding FP services were inadequate. The medical training during graduation or internship, and during the job, was found to be inadequate to provide quality FP services as per guidelines of nursing/medical council of India and Government of India on FP.

## Introduction

Family planning is one of the most critical components of a health service to ascertain effective sexual, reproductive and maternal health outcomes. Global estimates have shown that effective usage of contraception, can prevent 90% of maternal deaths related to unsafe abortions and 20% of overall obstetrics causes of mortality [1]. This would contribute in achieving the sustainable development goal of reducing the maternal mortality ratio to 70 per 100,000 live births by 2030, and goal 3.7 of ensuring universal access to sexual and reproductive health including family planning services [2]. However, providing universal access to family planning services requires that family planning workforce (specialist doctors, non-specialist doctors, registered and practitioner nurses, auxiliary nurse midwives, pharmacists, community health workers, etc.) are competent and possess necessary skills to deliver quality family planning services [3]. It has been documented that family planning services remained a neglected area in many countries, especially in the low and middle-income countries with high unmet need for family planning [4,5]. For instance, even though the family planning (FP) program was the first national program in India that started in the year 1952, it is the second most populous country in the world [6].

FP 2020 is a global partnership between 69 countries to empower women and girls by investing in rights-based FP [7]. India committed to FP 2020 in 2012 with an aim to provide choice, access, and quality of family planning services [8]. Since then India has expanded the range and reach of contraceptive options by introducing modern and more effective contraceptive methods and ensuring the availability at all levels of health care delivery system. Currently, FP has been integrated with Reproductive, Maternal, Newborn, Child, and Adolescent Health (RMNCH+A) strategy under the National Health Mission [9]. FP services are being provided free of cost at over 180000 primary level facilities, more than 6000 secondary level facilities, and over 750 tertiary level facilities, supported by a large pool of accredited private health facilities [10]. In addition to this, the country’s pool of community health workers like accredited social health activists (ASHAs, n = 900,000) are acting as depot holders for contraceptives, urine pregnancy kits at the village level and ensure adequate spacing between two births. Nurses working in the hospitals and certified auxiliary nurse midwives working at the peripheral health centers are allowed to prescribe the spacing methods like intra-uterine contraceptive devices (IUCDs), injectable contraceptives, oral contraceptive pills (OCPs), condoms and emergency contraceptive pills in addition to the counselling and consenting services. Doctors are trained to prescribe all these spacing methods of contraception as well as perform Minilap, Laparoscopic Sterilization and No-Scalpel Vasectomy (NSV) procedures for permanent sterilization [11,12].

Despite the concerted efforts, only a minimal improvement has been observed in the family planning (FP) indicators in India. The birth rate had declined from 23.8/1000 mid-year population in 2005 to 20.2/1000 mid-year population in 2017, total fertility rate from 2.7 to 2.2, contraception use rate declined from 56.3% to 53.5%, and unmet need of FP decreased from 13.9 to 12.9[13,14]. One will observe state wise disparities regarding various FP indicators. The total fertility rate varies from 3.41% in Bihar state to 1.17% in Sikkim state. Similarly, contraceptive use rate ranges from 25.0% in Manipur state to 89.5% in Chandigarh (Union Territory). Unmet need for contraception also varied from 22.3% in Nagaland to 4.6% in Andhra Pradesh [14]. Numerous reasons have been cited to explain the slow pace of progress in improvement of FP indicators in India, like the unmet need for contraception, unplanned pregnancy, early marriage and childbearing, low health providers to population ratio, untrained providers, poor quality of available services, irregular supplies, cultural or religious opposition, gender-based barriers and users and providers bias [8,15,16]. There is evidence that the cafeteria approach of providing FP services, where all the available contraceptive methods are offered to the clients with the list of side effects, benefits, and failure rate of each method so as to enable the clients to choose from the menu as per their need and preferences, is not being implemented effectively [8,17]. This is corroborated by the national family health survey data, round 4 (2015–16), that depicts female sterilization as the commonest contraceptive method used in India [14]. It may be the case that the service providers are fixed in their minds to provide only certain contraceptive methods to a set of clients (like offering only female sterilization for a woman who has completed her family with 2/3 living children), rather than offering her full basket of choices [18]. It may also be due to linkage of the financial incentives to the service providers if the permanent methods or long acting reversible contraceptives are chosen [19]. Uninformed choices lead to unintended pregnancies, lower contraceptive acceptance, and sterilization regrets, which is also a breach of reproductive rights of women [20]. Barriers that impede access to adequate contraceptives are insufficient number of service providers including the doctors and nurses, inadequate knowledge and skills of the service providers about the family planning services and reproductive rights of the women, poor quality of in-service trainings, and less emphasis on competency and skill-based learning during preservice medical education [21]. Improved quality of care has a positive correlation with sustained contraceptive usage by the women and meeting the unmet need [22]. It is, therefore, pertinent for the service providers to have adequate knowledge and skills about the services that they are offering and the reproductive rights of the women[23].

As per the Medical Council of India, it is necessary for the medical graduates to undergo a compulsory one-year rotatory internship after the final year to acquire the essential skills required as a doctor that includes delivering FP services and ensuring reproductive rights of the clients. Similarly, Indian Nursing Council ensures uniform standards of training for nurses, including general midwifery and nursing (GNM), auxiliary midwifery, and nursing (ANM) and Bachelor of Science in Nursing (BSc). They are trained for various FP services skills like counseling of the patients, consent taking, contraceptive prescription and usage techniques including IUCD insertion, condom usage, use of oral contraceptive pills, depot medroxy progesterone acetate (DMPA) injections etc. However, it has been observed that teaching practices in medical and nursing colleges in India are often not evidence-based, and may not align with ever-evolving standard protocols, the national guidelines concerning FP services, or web based Family Planning Training Resources [24–26]. There is a national training family planning plan for India, however, this is mainly implemented in the public health care delivery system, and medical colleges are generally left out [18]. The faculty in the medical colleges mostly teach from the standard textbooks and rarely use national level guidelines for teaching. As a result, there is a gap in the quality of FP services delivered by the service providers from the supply side of the chain and meeting the need of these services by the clients from the demand side. Unless we know the extent of this gap and its domains, it will be difficult to intervene effectively. With this background, this study was conducted with the objectives to assess the knowledge and skills of medical interns and nurses regarding FP services and reproductive rights of the women; and to document the prevailing FP practices in the teaching medical facilities in India, so that medical education could be strengthened, and family planning services could be improved.

## Methodology

### Study area

A cross-sectional study was conducted in three purposively selected states of India, including Delhi, Rajasthan, and Maharashtra, between November and December 2017. As per the census of India 2011, Maharashtra and Rajasthan are amongst the top 10 most populous states of India. As per the fourth round of the National Family Health Survey (2015–16), Rajasthan had low sex ratio at birth (females per 1,000 males) of 887, the infant mortality rate of 41 per 1,000 live births. The quality of FP services being offered is assessed by the facts that the contraceptive usage was 59.7% and an unmet need for FP amongst the currently married women aged between 15–49 years was 12.3% (spacing methods 5.7%). About 17.5% non-users of contraceptive methods reported that health workers had ever spoken to them about contraceptives. One of the job responsibilities of Auxiliary Nurse Midwives/female accredited social health activists workers in their field area is to counsel the couples who are not using any contraceptives, and thus motivating them to use/adopt any of the available contraceptive methods. The service providers are supposed to counsel the clients of family planning services about the possible side effects of the contraceptive methods except standard days method/lactational amenorrhoea. Nearly 56.5% of current users reported that they were never told about the side effects of the current method[14]. In Maharashtra, the sex ratio at birth (females per 1,000 males) was 924, the infant mortality rate of 24 per 1,000 live births. The contraceptive usage was 64.8% and an unmet need for FP amongst the currently married women aged between 15–49 years was 9.7% (spacing methods 5.3%). Only 18.5% non-users of contraceptive methods reported that health workers ever talked about family planning to them. About 36.3% of current users said that they were told about the side effects of the current method [18]. While in Delhi, the sex ratio at birth (females per 1,000 males) was 812, the infant mortality rate of 31 per 1,000 live births. The contraceptive usage was 54.9% and an unmet need for FP amongst the currently married women aged between 15–49 years was 15.0% (spacing methods 3.1%). Only 12% of non-users of contraceptive methods reported that health workers ever talked about family planning to them. Nearly 59.2% of current users reported that they were never told about the side effects of the current method[27,28].

### Study participants

The study participants were medical interns who had passed the final exam of bachelor of medicine and surgery (MBBS), and completed compulsory rotatory training in the department of obstetrics and gynecology; nurses, who were already in the job, with less than 5 years' of experience in the medical college or health posts attached with the medical colleges; and faculty or medical officer-in-charge of FP centre in the department of obstetrics and gynaecology or community medicine.

### Sample size and sampling technique

Since no previous information regarding prevalence was available, therefore as a thumb rule [29], correct knowledge percentage score of medical interns and nurses regarding FP services was assumed to be 50%, absolute precision as 10% and a design effect of 1.5, the sample size was calculated to be 145 using OpenEpi, version 3, open source calculator [30]. Assuming a 10% non-response rate, the final sample size is estimated to be 160 (80 interns and 80 nurses). Multistage simple random sampling technique was used to first select two districts within each study states, and then two medical colleges from the selected districts in the second stage. Private medical colleges were excluded, as it was difficult to get permission from these colleges. Since Delhi did not have districts, two medical colleges were selected randomly within it. However, the approval to conduct the study in one of the medical colleges could not be obtained. Hence, the additional medical college from Maharashtra was selected. In total six medical colleges (one medical college in Delhi, two in Rajasthan and three in Maharashtra) were included in the study. Interns who had fulfilled the inclusion criteria were enlisted, and proportionate number (19, 31 and 31 interns from Delhi, Rajasthan, and Maharashtra) were randomly selected. As per MCI guidelines an intern must have dedicated 15 days posting in family planning clinic[31]. Permission to interview nurses could not be obtained from the selected medical college in Delhi. Thirty two nurses from Rajasthan and 50 from Maharashtra were randomly selected.

Prior permission from the respective Dean of the selected medical colleges was obtained who liaised with the project team (a faculty and two postgraduate doctors) and with the faculty coordinating the duties of the interns and nursing superintendent in the respective medical college. The team obtained the list of the interns and nurses as per the eligibility criteria of the study. From this list, the required number of interns and nurses were randomly selected. The list of chosen nurses and interns were given appointments for the interview in a designated place as provided by the coordinating faculty/nursing superintendent of the medical college. After ensuring anonymity, written informed consent was obtained from the participants before the start of the interview.

### Study tools and data collection methods

The study tools were semi-structured and pretested. These were of two types, one dealt with assessing the knowledge and skills of the interns and nurses regarding family planning methods; and also about the training they have received during their graduation **(S1 File)**; and the second study tool was used to assess the prevailing FP practices in the department of obstetrics and gynecology. **(S2 File)**. The first tool had three parts including, a) background information of the participants and knowledge-based questions on contraceptive methods; b) skill assessment using an observation checklist based upon Objective Structured Clinical Examination (OSCE) for demonstration of steps of the intrauterine contraceptive device (e.g., CuT) insertion in a model, condom use on the thumb and use of Medical Eligibility Criteria (MEC) Wheel; and c) FP teaching and observation of training facilities. Information on part a) and b) of the first study tool was obtained from the interns and nurses, and information on part c) was obtained from the faculty of either obstetrics and gynecology or community medicine by face to face interview. **(S1 File)**. The information in the second tool was obtained by observing the family planning clinic regarding privacy (visual and auditory) of the clients, record review and stock position of contraceptive methods, and interview of the faculty/medical officer in charge of the FP clinics regarding FP practices. **(S2 File)**.

The study tools were developed in consultation with family planning experts from the United Nations Population Fund (UNFPA), faculty from the department of obstetrics and gynecology, department of community medicine and school of public health in Postgraduate Institute of Medical Education and Research (PGIMER), Chandigarh. The content validity was further established by another group of experts from the department of obstetrics and gynecology, PGIMER, Chandigarh. The tools were pretested in the government multi-specialty hospital, sector 16, Chandigarh, among 10% of the total sample size (n = 17) including interns (n = 7) and nurses (n = 10). It was realized during pretesting of the tool that nurses understand the questions better if they were interviewed in Hindi as compared to the English language, while there was no such issue with the interns, as English was the primary language of teaching in the medical colleges for the interns. Hence, the tool was translated into Hindi **(S3 File)** for the nurses as per the World Health Organisation's translation methodology.[20]

Two postgraduate doctors (Doctor of Medicine in Community Medicine and Masters in Public Health) were specially recruited, and trained by the experts (faculty) from obstetrics and gynecology and community medicine department to collect the data. The training of these doctors was done for 15 days, and it aimed at refreshing their knowledge and skills about the various family planning methods, the latest government of India guidelines on contraceptive methods and how to assess the skills as per OSCE. They were also sensitized about the differences in the FP services delivered by different FP workforce (nurses and doctors). Data collection was regularly supervised by the experts in the study states to ensure data quality.

### Data analysis

The responses obtained in different questions were assessed by assigning marks to each part of the question. The marking strategy for individual question is available as supplementary file. **(S4 File)**. Full and partial marks were given to the participant on the basis of answers provided by them. Full marks depicted that the participant was able to answer all the points of the answer, partial means that they answered only some parts of an answer, and wrong means that they did not give correct response. Data thus obtained was categorised into ordinal variables. Data was entered and analyzed in Statistical Package for Social Sciences, version 16.0. Proportions were estimated, and differences in proportions between interns and nurses regarding knowledge and skills in respect to use of various contraceptive methods, FP training received were considered significant at a 95% confidence interval. Since we did not estimate the sample size to compare the knowledge and skills of interns/nurses among the three states, hence we did not perform the state wise comparison.

### Ethical considerations

Ethical approval was obtained from the primary institute i.e. PGIMER, Chandigarh, India, to conduct the study in all the medical colleges. (PGI/IEC/2017/578). In addition, ethical approval was obtained individually from ethics committee/institutional review board of two other medical colleges including Grant Medical Colleges, Mumbai and Byramjee Jeejeebhoy Government Medical College and Sassoon General Hospitals, Pune. In the remaining four medical colleges, prior approval of the Head of institution was obtained.

## Results

A total of 81 interns and 82 nurses were enrolled in the study from Rajasthan (31 interns, 32 nurses), Maharashtra (31 interns, 50 nurses) and Delhi (19 interns). All the interns who were interviewed had completed their clinical postings in the department of Obstetrics and Gynaecology. At the time of interview, 74 (91.4%) interns were posted in community medicine department, 3 (3.7%) in surgery and 4 (4.9%) in paediatrics department in their respective medical colleges; and 55 (67%) nurses were posted in the obstetrics and gynaecology department, 24 (29.2%) in surgery (n = 24, 29.2%) and one (1.2%) in intensive care unit. All the nurses had a Bachelor level of qualification i.e. BSc Nursing (n = 30) or GNM (n = 52). Mean (standard deviation) age of interns were 23.8 (±1.2), and nurses 29.2 (±1.2) years. Males (50.6%) and females (49.3%) were equally represented among interns, while females (66.8%) were more among nurses. **(Table 1).**

**Table 1:** Background characteristics of study participants.

Characteristics	Interns n = 81 (%; 95% CI)	Nurses n = 82 (%; 95% CI)	Total n = 163 (%; 95% CI)	P value
**Sex**				
Male	41 (50.6; 45.0–56.2)	13 (15.8;12.2–19.4)	54 (33.1;29.4–36.8)	0.593
Female	40 (49.3; 43.7–54.9)	69 (84.1; 78.3–89.9)	109 (66.8;63.1–70.5)	
**Mean Age (standard deviation)**	23.8(1.2)	29.2(3.3)	26.4(3.7)	
**Age group**				
20–24	66 (81.5; 77.2–85.8)	4 (4.9; 3.1–6.7)	70 (42.9;39.0–46.8)	0.000
24–29	15(18.5; 14.2–22.8)	45 (54.9; 48.4–61.4)	60(36.8;33.0–46.8)	
30–34	0	30 (36.6; 30.6–42.6)	30(18.4;15.4–21.4)	
> = 35	0	3 (3.6; 1.5–5.7)	3(1.8;0.8–2.8)	
**Years of experience**				
< = 1 year	81 (100; 100–100)	2 (2.4; 1.2–3.6)	83(50.9;47.0–54.8)	0.000
2 years	0	22 (26.8;21.5–32.1)	22(13.5;10.8–16.2)	
3 years	0	16 (19.5;14.9–24.1)	16(9.8;7.5–12.1)	
4 years	0	24 (29.3;23.8–34.8)	24(14.7;11.9–17.5)	
> = 5 years	0	18 (22.0;17.1–26.9)	18(11.0;8.5–13.5)	
**Marital status**				
Married	7 (8.6;5.5–11.7)	70 (85.4)	77 (47.2;43.3–51.1)	0.000
Unmarried	74 (91.4;88.3–94.5)	12 (14.6)	86 (52.8;48.9–56.7)	
**State**				
**Delhi**				
* MC1	19 (23.5;18.8–28.2)	0	19 (11.7;9.2–14.2)	
**Rajasthan**				
MC 2	16 (19.8; 15.4–24.2)	16 (19.8; 15.4–24.2)	32 (19.6;16.5–22.7)	
MC 3	15 (18.5;14.2–22.8)	16 (19.8;15.4–24.2)	31 (19.0;15.9–22.1)	
**Maharashtra**				
MC 4	11 (13.6;9.8–17.4)	19 (23.5;18.8–28.2)	30 (18.4;15.4–21.4)	
MC 5	17 (21.0;16.5–25.5)	23 (28.4;23.4–33.4)	40 (24.5;21.1–27.9)	
MC 6	3 (3.7;1.6–5.8)	8(9.9; 6.6–13.2)	11 (6.7;4.7–8.7)	

* MC: Medical College; p value indicate the statistical significant difference in the proportion of interns and nurses

### Knowledge of interns and nurses regarding contraceptive methods

About 60% of the interns and 48% of the nurses could enumerate more than 5 contraceptives from a total of nine methods that are being offered by the government of India through the cafeteria approach including the Spacing Methods; *IUCD 380 A and Cu IUCD 375*, Injectable Contraceptive Depot Medroxy Progesterone Acetate *(Antara Programme)*, Combined Oral Contraceptive Pill *(Mala-N)*, Centchroman non-steroidal pill *(Chhaya)*, Progesterone-Only Pill *(POP)*, Condoms *(Nirodh)*, Emergency Contraceptive pills; and permanent sterilization methods including female Sterilization (tubectomy/tubal ligation: *laparoscopic*, *Minilap)* and male sterilization (no-scalpel vasectomy) [12,18]. Majority of the respondents thought that condoms (88.3%) and oral contraceptive pills (OCPs) [77.9%] were the best contraceptives for newly married couples. **(Table 2).** For a woman with one child, intrauterine contraceptive device (IUCD) [93.4%], and a woman with three children, sterilization (95.8%) was the most common response. About one-fifth of the participants (22%) responded that the contraceptives should not be given to a married woman who was coming alone. Nearly 31.9% (17.3% interns and 46.3% nurses) respondents told that it was illegal to provide the contraceptives to unmarried people.

**Table 2:** Knowledge of interns and nurses regarding various contraceptive methods and reproductive rights of the clients.

Parameters	Interns n = 81 (%; 95% CI)	Nurses n = 82 (%; 95% CI)	Total n = 163 (%; 95% CI)	p-value
Contraceptive choices for newly married couple*				
• Condom	74 (91.3; 88.2–94.4)	70 (85.3; 81.4–89.2)	144 (88.3; 85.8–90.8)	0.992
• OCP	68 (83.9; 79.8–88.0)	59 (71.9; 66.9–76.9)	127 (77.9; 74.7–81.1)	
• POP	6 (7.3; 4.4–10.2)	6 (7.4;4.5–10.3)	11 (6.8;4.8–8.8)	
• IUCD	30 (36.5; 31.2–41.8)	30 (36.5; 31.2–41.8)	60 (36.8;33.0–40.6)	
• All	1 (1.2; 0–2.4)	1 (1.2; 0.0–2.4)	2 (1.2;0.3–2.1)	
**Contraceptive choices for the woman with one child**				
• Condom	38 (46.9; 41.4–52.3)	44 (53.7; 48.2–59.2)	82 (50.3; 46.4–54.2)	0.460
• OCP	44 (54.3; 48.8–59.8)	47 (57.3; 51.8–62.8)	91 (55.8;51.9–59.7)	
• POP	3 (3.7; 1.6–5.8)	9 (11.0; 7.5–14.5)	12 (7.4;5.3–9.5)	
• IUCD	75 (92.6; 89.7–95.5)	77 (93.9; 91.3–96.5)	152 (93.4;91.5–95.3)	
• All	0	1 (1.2;0.0–2.4)	1 (0.6; 0.0–1.2)	
**Contraceptive choices for the woman with 3 children**				
• Condom	16 (19.8; 15.4–24.2)	21 (25.6;20.8–30.4)	37 (22.7; 19.4–26.0)	0.907
• OCP	15 (18.5; 14.2–22.8)	13 (15.8; 11.8–19.8)	28 (17.2; 14.2–20.2)	
• POP	1 (1.2; 0.0–2.4)	2 (2.4; 0.7–4.1)	3 (1.8; 0.8–2.8)	
• IUCD	48 (59.3; 53.8–64.8)	50 (61.0; 55.6–66.4)	98 (60.1; 56.3–63.9)	
• Sterilization	77 (95.1; 92.7–97.5)	79 (96.3; 94.2–98.4)	156 (95.8; 94.2–97.4)	
**Can contraceptives be given to a married woman coming alone?**				
• Yes	64 (79.0; 74.5–83.4)	35 (43.2; 37.7–48.7)	99 (61.1; 57.3–64.9)	0.000
• Yes, but after asking the family members	8 (4.9;2.5–7.3)	19 (11.7; 8.2–15.2)	27 (16.7; 13.8–19.6)	
• No	9 (11.1;7.6–14.6)	27 (33.3; 28.1–38.5)	36 (22.2;18.9–25.5)	
**Can contraceptives be given to unmarried women coming alone?**				
• Yes	60 (37.0; 31.6–42.4)	29 (17.9;13.7–22.1)	89 (54.9; 51.0–58.8)	0.000
• Yes but after asking the family members	7 (8.6;5.5–11.7)	11 (13.6; 9.8–17.4)	18 (11.1;8.6–13.6)	
• No	10 (12.4;8.7–16.1)	42 (51.9; 46.4–57.4)	52 (32.1; 28.4–35.8)	
**Is it legal to provide contraceptives to unmarried people**				
• Yes	52 (64.2; 58.9–69.5)	33 (40.2; 34.8–45.6)	85 (52.1; 48.2–56.0)	0.000
• No	14 (17.3; 13.1–21.5)	38 (46.3;40.8–51.8)	52 (31.9; 28.2–35.6)	
• Don’t know	15 (18.5;14.2–22.8)	11 (13.4; 9.6–17.2)	26 (16;13.1–18.9)	

*OCP: Oral Contraceptive Pill; POP: Progesterone Pills; IUCD: Intrauterine contraceptive devices; p value indicates the statistical significant difference in the proportion of interns and nurses regarding their knowledge.

Knowledge of interns and nurses regarding oral contraceptive pills, condoms, and emergency contraceptives is presented in **Table 3**. Respondents knew the common medical conditions like cardiovascular diseases (41.7%), breast diseases (30%), headache/migraine (25.7%) and thromboembolic disorders (22.1%) to rule out before prescribing OCPs.

**Table 3:** Knowledge of interns and nurses regarding oral contraceptive pills, condoms, and emergency contraceptives.

Contraceptives	Interns n = 81 (%; 95% CI)	Nurses n = 82 (%; 95% CI)	Total n = 163 (%; 95% CI)	p-value
**Oral contraceptives Pills (OCPs)**				
***Conditions to rule out from history before prescribing OCPs***				
• Smoking	10 (12.4; 8.7–16.1)	5 (6.1; 3.5–8.7)	15 (9.2; 6.9–11.5)	0.060
• Diabetes	10 (12.4;8.7–16.1)	15 (18.3; 14.0–22.6)	25 (15.3; 12.5–18.1)	
• Headaches	24 (29.6; 24.5–34.7)	18 (21.95; 17.3–26.5)	42 (25.7; 22.3–29.1)	
• Cardiovascular diseases	45 (55.6; 50.1–61.1)	23 (28.1; 23.1–33.1)	68 (41.7; 37.8–45.6)	
• Thromboembolic disorders	29 (35.8; 30.5–41.1)	7 (8.5;5.4–11.6)	36 (22.1; 18.9–25.3)	
• Post-partum haemorrhage	7 (8.6; 5.5–11.7)	2 (2.4; 0.7–4.1)	9 (5.5; 3.7–7.3)	
• liver disease	28 (34.6; 29.3–39.9)	11 (13.4;9.6–17.2)	39 (24.0; 20.7–27.3)	
• Breast disease	33 (40.7; 35.2–46.2)	16 (19.8; 15.4–24.2)	49 (30.6; 27.0–34.2)	
***Instructions to be given while prescribing OCPs should include***				
• When to start OCP	64 (79.0;74.5–83.5)	62 (76.6; 71.9–81.3)	126 (77.8; 74.5–81.1)	0.471
• Daily intake without fail	71 (87.7;84.1–91.3)	67 (82.7; 78.5–86.9)	138 (85.2; 82.4–88.0)	
• What to do if misses a pill	56 (69.5; 64.4–74.6)	41 (50.6; 45.1–56.1)	97 (59.9;56.1–63.7)	
• Possible side effects	22 (27.5;22.5–32.5)	13 (16.1; 12.0–20.2)	35 (21.6; 18.4–24.8)	
***What to do if the client misses 2 pills*?**				
• To take 2 pills the next day	42 (51.9; 46.3–57.5)	43 (53.1; 47.6–58.6)	85 (52.8; 48.9–56.7)	0.025
• Again 2 pills the second next day	16 (19.8; 15.4–24.2)	12 (14.9; 11.0–18.8)	28 (17.3; 18.4–24.8)	
• The couple should also use condom for 7 days	34 (42.0; 36.5–47.5)	12 (14.9; 11.0–18.8)	46 (24.4; 21.0–27.8)	
***Can OCPs be given to (yes)***				
• Newly married women	68 (84.0; 79.9–88.1)	43 (52.5; 47.0–58.0)	111 (68.1; 64.4–71.8)	0.079
• Illiterate women	64 (79.0; 74.5–83.5)	60 (73.2; 68.3–78.1)	124 (76.1; 72.8–73.6)	
• Women who do not want any more children	53 (65.4; 60.1–70.7)	61 (74.4; 69.6–79.2)	114 (70.0; 66.4–73.6)	
• Condoms				
***The failure rate of Condoms if used correctly***				
• <5%	34 (2.0; 0.4–3.6)	17 (21.0; 16.5–25.5)	51 (31.5; 27.9–35.1)	0.002
• 6–15%	35 (43.2; 37.7–48.7)	20 (24.7; 19.9–29.5)	55 (34.0; 30.3–37.7)	
• >15%	4 (5.0; 2.6–7.4)	5 (6.2; 3.5–8.9)	9 (5.6; 3.8–7.4)	
• Other	1 (1.2; 0.0–2.4)	6 (7.4; 4.5–10.3)	7 (4.3; 2.7–5.9)	
• Do not know	3 (3.7; 1.6–5.8)	12 (14.8; 10.9–18.7)	15 (9.3; 7.0–11.6)	
***Two most common Advantages of condom***				
• Dual protection against pregnancy and STI/HIV^	70 (86.4; 91.1–96.5)	47 (58.0; 52.5–63.5)	117 (72.2; 68.7–75.7)	0.016
• No side effects	23 (28.4;23.4–33.4)	34 (42.0; 36.5–47.5)	56 (35.2; 31.5–38.9)	
**Emergency contraceptives**				
***Type of contraception used after unprotected intercourse***				
• Emergency contraceptive pills	76 (93.8;91.1–96.5)	71 (86.6; 82.8–90.4)	147 (90.2; 87.9–92.5)	0.000
• Intrauterine contraceptive devices	45 (55.6; 50.1–61.1)	6 (7.3; 4.4–10.2)	51 (31.3; 27.7–34.9)	
• Yuzpe’s method	24 (29.6; 24.5–34.7)	4 (4.9; 2.5–7.3)	28 (17.2; 14.2–20.2)	
***The emergency contraceptive pill is effective if consumed within***				
• 24 hours of unprotected intercourse	0	2 (2.4; 0.7–4.1)	2 (1.2; 0.3–2.1)	0.177
• 48 hours of unprotected intercourse	3 (3.7; 1.6–5.8)	6 (7.32; 4.4–10.2)	9 (5.5; 3.7–7.3)	
• 72 hours of unprotected intercourse	77 (95.1; 92.7–97.5)	68 (82.9; 78.7–87.1)	145 (89.0; 86.5–91.5)	
**The frequency for taking centchroman**				
• 1 tablet weekly	18 (22.2; 17.6–26.8)	2 (2.4; 0.7–4.1)	20 (12.3; 9.7–14.9)	0.000
• Incorrect response	15 (18.5; 10.8–28.7)	0	15 (9.2; 5.2–14.7)	
• Do not know	48 (59.3; 53.8–64.8)	80 (97.6; 95.9–99.3)	128 (78.5; 75.3–81.7)	

*^STI/HIV*: *Sexually Transmitted Infections/Human Immunodeficiency Virus;* p value indicates the statistical significant difference in the proportion of interns and nurses regarding their knowledge.

Knowledge of interns and nurses regarding reversible long-acting contraceptives (IUCDs and hormonal contraceptives) and permanent contraceptives (tubectomy) is shown in **Table 4**. Duration of protection (10 years) offered by long-acting IUCD, i.e., CuT 380 A was known to 51.9% interns and 35.4% nurses. CuT 380 A is a T shaped intrauterine device which has a surface area of 380mm^2^ of copper, making it very effective with very low failure rate [32]. About 38% were aware that depot medroxyprogesterone acetate (DMPA). It is an injectable hormonal contraceptive that can be injected subcutaneously or in the muscles and is available free of cost in the government supply. About 83% of respondents had seen the eligibility checklist for tubectomy during their training period, but only half of them (43%) had seen tubectomy operation. Lactational amenorrhoea (LAM) means no menstruation among women in the post-partum period when they are breastfeeding (lactating) the baby. It has three essential components to be effective as a contraceptive method, including amenorrhea, exclusive breastfeeding, and six months of the postpartum period. This concept was clear to only 9.9% interns and 12.2% of the nurses.

**Table 4:** Knowledge of interns and nurses regarding reversible long–acting contraceptives (intrauterine contraceptive devices and hormonal contraceptives, post–partum contraception) and permanent contraceptives (tubectomy).

Parameters	Interns n = 81 (%; 95% CI)	Nurses n = 82 (%; 95% CI)	Total n = 163 (%; 95% CI)	P value
**Awareness about types of intrauterine contraceptive devices (IUCDs)**				
• Copper containing IUCDs	81 (100; 100–100)	78 (95.1; 92.7–97.5)	159 (97.6; 96.4–98.8)	0.000
• Hormonal IUCDs	60 (74.1; 69.2–79.0)	12 (14.6; 10.7–18.5)	72 (44.2; 40.4–48.2)	
• First generation /inert IUCDs	51 (63.0; 57.6–68.4)	23 (28.1; 23.1–33.1)	74 (45.4; 41.5–49.3)	
**Duration of protection offered by CuT 380 A**				
• 10 Years	42 (51.9; 46.3–57.5)	29 (35.4; 30.1–40.7)	71 (43.6; 39.7–47.5)	0.034
**Most common conditions to rule out before inserting Copper T**				
• Pregnancy	46 (56.8; 51.3–62.3)	40 (48.8;43.3–54.3)	86 (52.8; 48.9–56.7)	0.287
• STI/HIV	53 (65.4; 60.1–70.7)	36 (43.9; 38.4–49.4)	89 (54.6; 50.7–58.5)	
• Irregular Periods	50 (61.7; 56.3–67.1)	54 (65.9; 60.7–71.1)	104 (63.8; 60.0–67.6)	
• Adnexal Mass/Ectopic Pregnancy	41(50.6; 45.0–56.2)	25 (30.5; 25.4–35.6)	66 (40.5; 36.7–44.3)	
• Multiple Sexual Partners	1 (1.2; 0.0–2.4)	0	1 (0.6; 0.0–1.2)	
**Most common side effects of Cu-T insertion**				
• Pain/cramps	61 (75.3; 70.5–80.1)	55 (67.9; 62.7–73.1)	116 (71.6; 68.1–75.1)	0.004
• Bleeding/menorrhagia/spotting/irregular bleeding	61 (75.3; 70.5–80.1)	72 (88.9; 85.4–92.4)	133 (82.1; 79.1–85.1)	
• Infections/PID/vaginal discharge	51 (63.0; 57.6–68.4)	32 (39.5; 34.1–44.9)	83 (51.2; 47.3–55.1)	
• Expulsion	29 (34.6; 29.3–39.9)	9 (11.1; 8.6–15.8)	37 (22.8; 19.5–26.1)	
**When is Post-Partum IUCD (PPIUCD) to be inserted**				
• Within 10 minutes of delivery	37 (45.7; 40.2–51.2)	30 (36.4; 31.1–41.7)	67 (41.1; 37.2–45.0)	0.001
• Within 48 hours	27 (33.3; 28.1–38.5)	10 (12.2; 8.6–15.8)	37 (22.7; 19.4–26.0)	
• During caesarean section	10 (12.4; 8.7–16.1)	8 (9.8; 6.5–13.1)	18 (11.0; 8.5–13.5)	
• Other	26 (32.1; 26.8–37.2)	11 (13.4; 9.6–17.2)	37 (22.7; 19.4–26.0)	
• Don’t know	2 (2.5; 0.8–4.2)	13 (16.0; 12.0–20.0)	15 (9.3; 7.0–11.6)	
**Time for taking consent for PPIUCD insertion**				
• Ante-natal period	40 (49.4; 43.8–55.0)	29 (35.8; 30.5–41.1)	69 (42.3; 38.4–46.2)	0.001
• Inta-natal period	31 (38.3; 32.9–43.7)	16 (19.5; 15.1–23.9)	45 (28.8; 25.3–32.3)	
• Post-natal period	8 (9.9; 6.6–13.2)	25 (30.9; 25.8–36.0)	33 (20.4; 17.2–23.6)	
**Questions to ask before inserting Depot Medroxy Progesterone Acetate (DMPA)**				
• Pregnancy	14 (17.3;13.1–21.5)	12 (14.8; 10.9–18.7)	26 (16.0; 13.1–18.9)	0.639
• Irregular periods	24 (29.6; 24.5–34.7)	15 (18.5; 14.2–22.8)	39 (24.7; 21.3–28.1)	
• Breast cancer	20 (24.7; 19.9–29.5)	12 (14.9; 11.0–18.8)	32 (19.8; 16.7–22.9)	
• Liver diseases	9 (11.1; 7.6–14.6)	5 (6.2; 3.5–8.9)	14 (8.6; 6.4–10.8)	
• Thromboembolic episodes	11 (13.6; 9.8–17.4)	7 (8.6; 5.5–11.7)	18 (11.1; 8.6–13.6)	
**Topics to be covered while counseling for DMPA**				
• Menstruation related side effects	27 (33.3; 28.1–38.5)	14 (17.2; 13.0–21.4)	41 (25.2; 21.8–28.6)	0.668
• Delayed return of fertility	14 (17.3; 13.1–21.5)	5 (6.1; 3.5–8.7)	19 (11.6; 9.1–14.1)	
• Don’t know	19 (23.7; 19.0–28.4)	12 (15.0; 11.1–18.9)	31 (19.4; 16.3–22.5)	
**Contraceptives that can be advised to breastfeeding mother**				
• Intrauterine contraceptive device	45 (55.6; 50.1–61.1)	61 (74.4; 69.6–79.2)	106 (65.0; 61.3–68.7)	0.000
• Injectable contraceptives	8 (9.9; 6.6–13.2)	9 (11.0; 7.5–14.5)	17 (10.4; 8.0–12.8)	
• Progesterone only Pills	31 (38.3; 32.9–43.7)	6 (7.3;4.4–10.2)	37 (22.7; 19.4–26.0)	
• Condoms	56 (69.1; 64.0–74.2)	54 (65.9; 60.7–71.1)	110 (67.9; 64.2–71.6)	
**Tubectomy: Have ever seen (yes)**				
• Eligibility checklist for Tubectomy	12 (85.2; 81.3–89.1)	14 (79.3; 74.8–83.8)	26 (82.2; 79.2–85.2)	0.259
• Tubectomy operation	31 (38.3; 32.9–43.7)	39 (47.6; 42.1–53.1)	70 (43.0; 39.1–46.9)	
• Consent form for Tubectomy	24 (29.6; 24.5–34.7)	50 (61.0; 55.6–66.4)	74 (45.4; 41.5–49.3)	
**Prerequisites for lactational amenorrhea to be an effective contraceptive**				
• Amenorrhoea	18 (22.2; 17.6–26.8)	14 (83.0; 78.9–87.1)	32 (80.4; 77.3–83.5)	0.024
• Exclusive breast feeding	59 (72.8; 67.9–77.7)	45 (54.9; 49.4–60.4)	104 (63.8; 60.0–67.6)	
• 6 months post-partum	43 (53.1; 47.6–58.6)	36 (43.9; 38.4–49.4)	79 (48.5; 44.6–52.4)	
• All	15 (18.5; 10.8–28.7)	11 (13.4; 6.9–22.7)	26 (16.0; 10.7–22.5)	
• Don’t know	11 (13.8; 10.0–17.6)	27 (33.3; 1.3–5.3)	38 (23.6; 20.3–26.9)	

p value indicates the statistical significant difference in the proportion of interns and nurses regarding their knowledge.

Overall, the assessment of knowledge in terms of correct, partial, and wrong is presented in **Table 5**.

**Table 5:** Overall knowledge of interns and nurses regarding various contraceptive methods.

Parameters (Choices)*	The response of Interns n = 81 (%)				The response of Nurses n = 82 (%)				p-value
	Correct	Partially correct	Wrong	Don’t know	Correct	Partially correct	Wrong	Don’t know	
Contraceptive choices for newly married couple (Condom, OCP, POP, IUCD, all, few of these)	23 (28.3)	50 (61.7)	8 (9.8)	0	23 (28.0)	36 (43.0)	31 (37.8)	0 (0)	0.000
Contraceptive choices for a woman with 1 child (Condom, OCP, POP, IUCD, all, few of these)	27 (33.3)	28 (34.5)	26 (32.1)	0	35 (42.3)	23 (28)	23 (28.0)	1(1.2)	0.440
Contraceptive choices for the woman with 3 children (Condom, OCP, POP, IUCD, sterilization, all, few of these)	22 (27.1)	27 (33.3)	31 (38.2)	1 (1.2)	26 (31.7)	27 (32.1)	29 (34.1)	0 (0)	0.707
Can contraceptives be given to a married woman coming alone?	68 (83.9)	0	13 (16.0)	0	37 (45.1)	0	45 (54.8)	0 (0)	0.000
Can Contraceptives be given to unmarried women coming alone?	61 (75.3)	0	18 (22.2)	2 (2.4)	32 (39)	0	48 (58.5)	2 (2.4)	0.000
Is it legal to provide contraceptives to unmarried people?	52 (64.1)	0	14 (17.2)	15 (18.5)	33 (40.2)	0	38 (46.3)	11 (13.4)	0.000
Types of IUCDs available (1^st^ generation, Copper, Hormonal)	18 (22.2)	15 (18.5)	48 (59.3)	0	51 (62.2)	17 (20.7)	9 (11.0)	1 (1.2)	0.000
Common contraindications for IUCDs (pregnancy, STI/HIV, irregular periods/ adnexal mass, ectopic pregnancy, multiple sexual partners)	17 (21.0)	18 (22.0)	42 (51.9)	3 (3.7)	20 (24.4)	27 (32.9)	25 (30.5)	8 (9.8)	0.035
Common side effects of IUCDs (cramps, menstrual problems, infections, RTI/STI, expulsion)	12 (14.8)	31 (38.3)	36 (44.1)	1 (1.2)	11 (13.4)	38 (46.3)	26 (32.7)	5 (6.1)	0.169
Types of Cu-T in government supply (CuT375, 380A)	24 (29.6)	48 (59.3)	3 (3.7)	6 (7.4)	19 (23.2)	28 (34.1)	6 (7.3)	29 (35.4)	0.000
How long does Cu-T 380A offer protection?	42 (51.91)	0 (0)	34 (42.0)	5 (6.2)	29 (35.4)	0 (0)	37 (45.1)	16 (19.5)	0.016
When is PPIUCD inserted?	15 (18.5)	43 (53.1)	11 (13.6)	12 (14.8)	9 (11.0)	36 (43.9)	8 (9.8)	29 (35.4)	0.022
When is consent for PPIUCD taken?	61 (75.3)	0 (0)	10 (12.3)	10 (12.3)	3 (3.7)	43 (52.4)	17 (20.7)	19 (23.2)	0.000
Medical contraindications for OCPs (Any 4 correct responses)	21 (25.9)	0 (0)	49 (60.5)	11 (13.6)	15 (17.3)	0 (0)	35 (42.7)	32 (39.0)	0.001
Yes OCPs can be bought over the counter	53 (65.4)	0 (0)	24 (29.6)	4 (4.9)	52 (62.3)	0 (0)	29 (35.4)	1 (1.2)	0.321
Instructions were given before starting OCPs	48 (59.3)	27 (33.3)	4 (4.9)	2 (2.5)	38 (46.3)	32 (39.0)	5 (6.1)	7 (8.5)	0.215
Instructions if 2 pills are missed	10 (12.3)	29 (35.8)	20 (24.7)	22 (27.2)	4 (4.9)	25 (30.5)	22 (26.8)	31 (37.8)	0.214
Whom can OCPs be given to? (Newly married women, illiterate women, Women who do not want any more children)	41 (50.6)	26 (32.1)	14 (17.3)	0 (0)	31 (37.8)	28 (34.1)	22 (26.8)	1 (1.2)	0.237
Which OCPs are available in govt. supply (Mala N, Mala D)^	14 (17.3)	63 (77.8)	3 (3.7)	1 (1.2)	23 (28.0)	39 (47.6)	5 (6.1)	15 (18.3)	0.000
What kind of contraceptive is DMPA?	59 (72.8)	0(0)	8 (9.9)	14 (17.3)	32 (39.0)	0(0)	3 (3.7)	47 (57.3)	0.000
Medical contraindications for DMPA (Any 3 correct responses out of pregnancy/irregular periods, breast cancer, liver disease, thromboembolic episodes)	8 (9.9)	21 (25.9)	11 (13.6)	41 (50.6)	5 (6.1)	16 (19.5)	3 (3.7)	58 (70.0)	0.031
Common side effects of DMPA (menstruation related side effects, delayed return of fertility)	9 (11.1)	22 (27.2)	4 (4.9)	46 (56.8)	4 (4.9)	12 (14.6)	2 (2.4)	64 (78.0)	0.037
Yes, injectable contraceptives are available in the government supply	36 (44.4)	0	33 (40.7)	12 (14.8)	28 (34.1)	0	28 (34.1)	26 (31.7)	0.038
Prerequisites for lactational amenorrhea method	8 (9.9)	44 (54.3)	15 (18.5)	14 (17.3)	10 (12.2)	25 (30.5)	17 (20.7)	30 (36.6)	0.010
Contraceptives that can be given to breastfeeding women (Condoms, POP, IUCD, Injectable)	14 (17.3)	45 (55.6)	16 (19.8)	6 (7.4)	14 (17.1)	35 (42.7)	26 (31.7)	7 (8.5)	0.296

*OCP: *Oral Contraceptive Device; POP*: *Progesterone Only Pill; IUCD*: *Intrauterine Contraceptive Device; DMPA*: *Depot medroxy progesterone acetate*, *PPIUCD*: *Post–Partum Intrauterine Contraceptive Device; STI/HIV*: *Sexually Transmitted Infections/Human Immunodeficiency Virus;*

^ *Mala–N is a Low dose OCP with nor–ethisterone acetate and ethinyloestradiol and is available in Government supply*, *while Mala–D is also Combined OCP with levonorgestrel and ethinyloestradiol and is available at the cost of INR 3 under social marketing*; p value indicates the statistical significant difference in the proportion of interns and nurses regarding their knowledge.

Gender wise assessment was done to analyse differences in knowledge between male and female participants, including interns and nurses, and presented in **S1 Table.** Though, proportion of females was higher with correct responses, but statistical significance could be seen only for a few questions specifically. Overall, females had better knowledge (statistically significant) pertaining to reproductive rights. However, female interns had significantly better knowledge as compared to male interns regarding the choice of contraceptives for women with one child (47.5% vs. 24.4%; p = 0.036), with three children (45% vs. 17.1%; p = 0.042); and regarding types of IUCDs (90% vs. 65.9%; p = 0.009). Older participants **(>25 years)** had better knowledge regarding common conditions to be ruled out before inserting CuT (52% versus 30%;p-value: 0.016), OCP prescription(20% versus 15%;p-value: 0.001), instructions to be given to patients (63%% versus 44%;p-value: 0.011), emergency contraception (31% versus 10%;p-value: 0.000), frequency of taking centchroman (21% versus 2%; p-value: 0.00), etc. Younger participants (≤ 25 years) responded better to knowledge based questions **(S2 Table).** However, married participants depicted better knowledge (statistically significant with p value <0.05) in certain areas pertaining to reproductive rights (41% and 35% for married. versus 6% and 6% for unmarried participants.) and legality of contraceptives to be given to unmarried people (39% versus 4%;p-value: 0.000). It was observed that this association significantly (p = 0.049) improved with age among nurses, as 65% of the nurses between 30–34 years have answered it correctly as compared to 32.7% and 28.6% among 25–29 years and 20–24 years age group, respectively. **(S3 Table)**

### Skills of nurses and interns regarding the use of contraceptive methods (objective structured clinical examination)

About 19.8% interns and 64.6% nurses refused to demonstrate the use of MEC wheel for choosing the best contraceptives for hypothetical cases, as shown in **Table 6**. Further, 43.2% interns and 69.5% nurses refused, while 12.2% nurses and 44.4% interns passed in demonstrating CuT insertion in dummy uterus. Exact steps of using a condom on the thumb were demonstrated by 63% interns and 40.2% nurses.

**Table 6:** Objective structured clinical examination (OSCE) score of interns and nurses.

Parameters	Interns n = 81 (%)	Nurses n = 82 (%)	p-value
**Medical eligibility criteria (MEC) wheel demonstration**			
• Pass	57 (70.4; 65.3–75.5)	22 (26.8; 21.9–31.7)	0.148
• Fail	8 (9.9; 6.6–13.2)	7 (8.5; 5.4–11.6)	
• Refused to demonstrate appropriate usage of MEC	16 (19.8; 15.4–24.2)	53 (64.6; 59.3–69.9)	
**Intrauterine contraceptive device (IUCD): Cu T**			
• Pass	36 (44.4; 38.9–49.9)	10 (12.2; 8.6–15.8)	0.000
• Fail	10 (12.3; 8.7–15.9)	15 (18.3; 14.0–22.6)	
• Refused to perform a demonstration of Cu T insertion	35 (43.2; 37.7–48.7)	57 (69.5; 64.4–74.6)	
**Steps for CuT insertion**			
• Washes hands and wears gloves	31 (38.3; 32.9–43.7)	15 (18.3; 14.0–22.6)	0.729
• Insert the sterile sound with ‘no touch’ technique	38 (46.9; 41.4–52.4)	12 (14.6; 10.7–18.5)	
• Load IUCD in its sterile package	31 (38.2; 32.8–43.6)	7 (8.6; 5.5–11.7)	
• Set the blue depth gauge to the measurement of the uterus	29 (35.8; 29.9–40.5)	9 (11.0; 7.5–14.5)	
• Carefully insert the loaded IUCD and release it into the uterus	40 (49.4; 43.8–55.0)	14 (17.1; 12.9–21.3)	
• Take out the plunger	31 (38.3; 32.9–43.7)	15 (18.3; 14.0–22.6)	
• Partially withdraw the insertion tube till the string are visible	24 (29.6; 24.5–34.7)	9 (11.0; 7.5–14.5)	
• Use sterile scissors to cut the IUCD strings to 3–4 cm length in the vagina	20 (24.7; 19.9–29.5)	11 (13.4; 9.6–17.2)	
**Barrier contraceptive usage (Condom)**			
• Pass	51 (63.0; 57.6–68.4)	33 (40.2; 34.8–45.6)	0.348
• Fail	15 (18.5; 14.2–22.8)	12 (14.6; 10.7–18.5)	
• Refused to perform demonstration of Condom usage	15 (18.5; 14.2–22.8)	37 (45.1; 39.6–50.6)	
**Steps for condom usage**			
• Check the expiry date on the wrapper	1 (1.2; 0.0–2.4)	3 (3.7; 1.6–5.8)	
• Open the package without tearing the condom	64 (79; 74.5–83.5)	42 (51.2; 45.7–56.7)	0.985
• Do not use teeth to open the wrapper	64 (79.0; 74.5–83.5)	41 (50.0; 44.5–55.5)	
• Hold the condom by the last ½ inch at the tip, making sure to squeeze out any air	31 (38.3; 32.9–43.7)	19 (23.2; 18.5–27.9)	
• Put the condom on the tip of the thumb.	61 (75.3; 70.5–80.1)	38 (46.3; 40.8–51.8)	
• While still pinching the tip, unroll the condom down the shaft, to the base	33 (40.4; 34.9–45.9)	27 (32.9; 27.7–38.1)	
• Remove the condom by rolling it off	51 (63.0; 57.6–68.4)	31 (37.8; 32.4–43.2)	
• Throw the condom into the dustbin	39 (48.1; 42.5–53.7)	24 (29.3; 24.3–34.3)	

p value indicates the statistical significant difference in the proportion of interns and nurses regarding their skills.

### Status of training of interns and nurses on family planning methods and services

Almost 90% of interns received training on FP methods and services. **(Table 7).** About 76.5% of interns reported that they were ever posted in FP clinic (for 15 days) during training. Although 63% of interns and nurses have seen the insertion of IUCD, more nurses (17.1%) reported performing IUCD insertion than interns (12.3%) at least once. A very small proportion of interns and nurses reported having seen implantable contraceptives (12.3%) and spermicides (17.3%).

**Table 7:** Status of training of interns and nurses on family planning (FP) methods and services (as reported by the participants).

	Interns n = 81 (%)	Nurses n = 82 (%)	p-value
Status of Training*			
• Attended a class on FP methods during training	73 (90.1; 86.8–93.4)	64 (78.0; 73.4–82.6)	0.035
• Ever Posted in FP clinic	62 (76.5; 71.8–81.2)	38 (46.3; 40.8–51.8)	0
• Observed counseling on different FP methods	48 (59.3;53.8–64.8)	57 (69.5; 64.4–74.6)	0.172
• Ever seen MEC wheel	10 (12.3; 8.7–15.9)	6 (7.3; 4.4–10.2)	0.281
• Ever observed MEC wheel used for a patient	6 (7.4; 4.5–10.3)	4 (4.9; 2.5–7.3)	0.501
Number of students who have ever*			
• Observed IUCD insertion	51 (63.0; 57.6–68.4)	52 (63.4; 58.1–68.7)	0.952
• Performed IUCD insertion	10 (12.3; 8.7–15.9)	14 (17.1; 12.9–21.3)	0.394
• Inserted IUCD on dummy/ model	9 (11.1; 7.6–14.6)	11 (13.4; 9.6–17.2)	0.645
• Observed IUCD removal	26 (32.1; 26.9–37.3)	42 (51.2; 45.7–56.7)	0.013
• Performed IUCD removal	16 (19.8; 15.4–24.2)	23 (28.0; 23.0–33.0)	0.215
**Number of students who have ever seen**			
• Condom	78 (96.3; 94.3–98.4)	82 (100; 100–100)	0.079
• Oral Contraceptive Pill	78 (96.3; 94.2–98.4)	80 (97.6; 95.9–99.3)	0.64
• Intrauterine Device	80 (98.8; 97.6–100)	81 (98.8; 97.6–100)	0.993
• Depot medroxy progesterone acetate	32 (39.5; 34.1–44.9)	28 (34.1; 28.9–39.3)	0.478
• Emergency Contraceptive Pill	64 (79.0; 74.5–83.4)	63 (76.8; 72.1–81.5)	0.737
• Centchroman	29 (35.8; 30.5–41.1)	19 (23.2; 18.5–27.9)	0.077
• Hormonal IUCD	38 (46.9; 41.4–52.3)	9 (11.0; 7.5–14.5)	0
• Implantable Contraceptives	10 (12.3; 8.7–15.9)	13 (15.9; 11.9–19.9)	0.52
• Spermicides	14 (17.3; 13,1–21.5)	5 (6.1; 3.5–8.7)	0.026

*MEC: Medical Eligibility Checklist; IUCD: Intrauterine contraceptive device; p value indicates the statistical significant difference in the proportion of interns and nurses

All the medical colleges reported that they provide adequate training to their students pertaining to the FP methods and services. The teaching practices were assessed in all the medical colleges (n = 6) that involved infrastructure appraisal, teaching aid and material. It was observed that department of Community Medicine (n = 3) and department of Obstetrics and Gynaecology (n = 3) were jointly involved in teaching family planning services to the students. (**S4 Table)**. Dedicated family planning rooms were available in four medical colleges. Only two colleges had FP skill labs and dummies for demonstrations, while only two medical college had models for family planning courses. However, samples of most of the contraceptives in provided under the government supply (Oral pills, Condoms, Injectable, Emergency contraceptives and Cu T 380 A/375) were available in majority of the medical colleges (N = 5). Only one college had a medical eligibility checklist (MEC) wheel for training purposes. Only two colleges were using the updated national guidelines issued by the government of India for teaching purposes, while others preferred only the standard textbooks. All the colleges stated that they provided hands on trainings to their students.

### Status of prevailing family planning practices

Family planning clinics in all the medical colleges (n = 6) were assessed for the prevailing practices (**S5 Table**). It involved observation of the family planning services that included counselling of clients, assessment of clients eligibility for various family planning methods opted by them. It was observed that auditory privacy was available in only four medical colleges, while MEC wheel was being used only in one medical college. None of the college had MEC wheels in sufficient numbers. Records of these clinics were assessed for stock position of contraceptives and the number of person who were provided FP services in last month. Adequate stock of FP methods (contraceptives, pregnancy test, CuT, etc.) was available in almost all the colleges. However, only two medical colleges were offering vasectomy services, while two colleges had injectables and emergency contraceptives in their stocks. Three medical colleges reported to have an established FP logistic management information system.

## Discussion

This study highlights the inadequate knowledge and skills of the interns (pre service cadre) and nurses (in-service cadre) regarding family planning services in the medical colleges in Rajasthan, Maharashtra, and Delhi, India. The status of preservice medical education and prevailing practices regarding FP services was also found to be inadequate. These results have many implications not only in providing quality FP services at present but also in terms of producing skilled FP workforce in the future. It may also adversely affect the universal access to quality reproductive and sexual health to the clients, which may lead to a compromise in the reproductive rights of the population, especially of the women, as they are most often not the decision makers in opting for the FP methods. The findings of this study are similar to family planning training assessment studies done in Maharashtra, India and Tanzania [33,34] It is reported that majority of the medical schools were unable to produce competent FP service providers, and the teaching was poorly guided, mainly theoretical with less practical exposure related to FP skill development.

The tool (S1 File) which we used had direct and open ended questions related to choice of contraceptive methods to be offered to the women as per their need, medical history to be ruled out before prescribing certain contraceptive methods like OCPs, DMPA, and questions related to the common side effects. Only the interviewer had the list of the answers as options available with them. We did not specifically asked the categories of the conditions to prescribe OCP/DMPA from the respondents. Options were based on government of India guidelines[18,35,36]. We only asked whether the interns or nurses would like to rule out certain medical conditions or not before prescribing these contraceptives. At their level, we expect them to know about the major conditions which should raise an alarm and they should be able to tell the patient to consult a specialist of obstetrics and gynaecology to advise further.

Our study sample had proportionate representation from the male (54, 33.1%) and female (109,66.9%) groups. A higher proportion of females were because the majority of the nurses were females (84.1%). Significantly higher knowledge of female participants in many questions as observed in this study is similar to Raselekoane NR (2016) study, who reported a non-serious approach of the male students towards contraception and family planning [37]. Gender differences should not be ignored during medical education to avoid compromise with the sexual and reproductive health rights of the clients [38]. Younger participants depicted better knowledge pertaining to various contraceptives. But specifically amongst nurses, improvement in knowledge in specific topics with age may be attributed to change in marital status. Married females tend to gain clarity of certain concepts regarding FP methods as they are free to use and discuss them more often than their unmarried counterparts. It is corroborated by the fact that there is a notable difference in contraceptive usage and family planning practices based upon the women’s marital status in developing countries like India [39].

It is recommended to hold in-service refresher training programs as a routine activity in the health system. This can be done under the trainings held under reproductive maternal neonatal, child and adolescent health strategy under National Health Mission in India with inclusion of nurses working in the medical colleges [40]. As per the guidelines, there is a provision of capacity building of the services providers for family planning services, especially onsite training especially for intrauterine devices by organizations like Johns Hopkins Program for International Education in Gynecology and Obstetrics (Jhpiego), Engender Health, International Projects Assistance Services (IPAS) [18]. However, medical colleges may deny to be trained by the external organizations, or may give an excuse of limited funds for such purposes. However, such events could be planned during drafting of the state program implementation plan or medical colleges can plan as part of continued nursing or medical education annually with their own or external expert faculty members.

It was observed that participants did not report to provide FP methods as per the cafeteria approach to an eligible woman [15]. Most of them were unaware of the new FP methods [like DMPA (86%)] that have been introduced in India [12]. Rhythm method (87.9%), coitus interrupts (55.4%), LAM (45.2%) were preferred natural methods during counseling. However, these are not promoted in newer family planning programs. Fehring RJ et al. (Milwaukee, 2001), also observed that when a client specifically asked for information on Natural Family Planning methods, 62.3%, 47.7%, 42.8% and 37% of the certified nurse-midwives (CNM) respondents would describe the symptom-thermal method, the ovulation method, the Basal Body Temperature method, and the calendar method, but did not recommend the use of these methods as a means of avoiding pregnancy [41]. In our study, IUCD, condom, and sterilization were preferred contraceptives for newly married women, for women with one child and women with three children. Jain AK et al. (India, 2017), observed 15.6% of contraceptive users receiving information on all four items (IUCDs, condom, sterilization, and pills) [42]. In our study, 32.1% of participants reported that FP services should not be offered to unmarried females coming alone, and 31.9% of participants reported that providing FP services to unmarried people is illegal. Tilhaun et al. study (Ethiopia, 2010) also observed a negative attitude toward providing FP methods to unmarried adolescents [43]. Sociocultural norms of Indian society contribute to having sex-related issues a taboo and hinder young people from seeking counseling regarding sexual health [44]. Though, there is no specific law against or in favour of prescribing contraceptives to unmarried people in India, but it has been reported earlier that that healthcare providers often impose unnecessary barriers in dispensing contraceptives, including denial of a contraceptive method based on age, parity, marital status or lack of parental or spousal authorization [45,46]. There are Family planning guidelines and adolescent reproductive and sexual health guidelines under National Health Mission, Ministry of Health and Family Welfare, Government of India [12] and WHO [47], which states that women in the reproductive age group (15 to 49 years) including adolescent girls irrespective of the marital status (married or unmarried) should be made aware about the contraceptive services; and these should be made accessible to them. There is no legal issue in offering contraceptives to the unmarried people.

Knowledge regarding side effects and contraindications of IUCD use (68.7%) was better than other studies done in the past [48,49]. This may be attributed to improvement in health promotion activities conducted in the last decade. We stress on the fact that the participants were asked to enumerate the conditions that should be ruled out before inserting Copper-T. Though HIV is not a direct contraindication, but the WHO medical eligibility criteria caution against IUD and LNG-IUS use by women at risk of HIV, HIV positive women, and women with AIDS. This is a grade 3 criterion “theoretical or proved risks generally outweigh the advantages” as opposed to grade 4 which is an “unacceptable health risk.” There are a number of concerns about IUD use by women with HIV infection relating to contraceptive efficacy, risks of sexual transmission, and acute pelvic inflammatory disease [50,51]. Regarding PPIUCD’s insertion, timing of consent taken was asked from the respondents to ascertain their knowledge regarding the reproductive rights of the women. (Table 4). As per the PPIUCD reference manual, counselling for informed consent should take place in the antenatal period, in early labor or immediately postpartum. Counselling for informed consent should not take place during the active phase of labor[36].

Few interns (12%) had adequate knowledge about instructions that should be given to a woman who had missed up to two doses of her OCPs. These findings are in line with a study by Rutter W et al. (Australia 1988) [52]. Adequate knowledge of interns and nurses regarding male condoms (72%) in this study is similar to the observations made by Simbar et al. study (Iran 2005) [53].

More than 60% of participants were unaware about injectable contraceptives, particularly DMPA. Hogmark et al. (India, 2013), reported that despite the positive attitude of medical students towards modern contraceptives, sex education and FP counseling, they still had misconceptions about modern methods of contraception in Maharashtra, which is consistent with the findings of the present study [33]. Knowledge about lactational amenorrhoea was partial, as only 9.9% interns and 12.2% nurses could correctly enumerate its three criteria (Table 4) to be an efficacious method [54]. Singh S et al. (Delhi, 2002) observed high awareness about emergency contraception among doctors who felt that the use of emergency contraceptives could bring down the number of induced abortions [55]. Less than half of the respondents (61% nurses; 30% interns) had ever witnessed tubectomy operation, while 16% had seen eligibility checklist, and 45.5% had seen the consent form for tubectomy. This difference is probably because taking consent from patients is primarily a job responsibility of nurses in India. Few interns and nurses accepted that they did not attend any family planning classes during their degree course. We did not look into the reasons behind low attendance during family planning classes. Anecdotally, it is a general notion amongst the medical students that there is no need to attend family planning classes if you don’t want to be a gynaecologist. However, this aspect needs further exploration because of its long term implications. Attendance can be improved with compulsory weightage to family planning during passing exams.

Availability of adequate infrastructure is an essential part of pre-service trainings and helps students to adhere to the standard protocols with the clients. In our study it was observed that only 1 out of 6 medical colleges had a MEC wheel. As these were not used in the routine FP practices in most of the medical colleges, hence students have not observed these during their training period and this is attributed as one of the major reasons behind their refusal to demonstrate the use of MEC wheel during OSCE. A similar study reported difficulties in delivering quality services as per the protocols, which were attributed to improper facility layout and lack of furniture [56]. Inadequate logistics and privacy may also affect the client's satisfaction [57,58].

One of the main strength of our study was the use of reliable and valid method, i.e., OSCE, to assess the skills of the study participants [59–61]. Some nurses (45.1%) refused to demonstrate the procedure of using a condom, which could be linked with shyness, or with unawareness of the correct process. This has implications on proper demonstration of condom usage to the clients, which may lead to higher failure rates and inconsistent use of condoms. This may also result in increased use of emergency contraceptives in the population at high risk of unintended pregnancy. Lim MS et al. (China, 2015) has stressed that efforts should be made to sensitize the students to rise above and overcome this social taboo, and emphasis should be given to training in skills for condom/contraception negotiation, as partner refusal to use condoms is common [62]. The use of MEC wheel during pre-service training should be stressed upon as it aims to guide health-care providers in decision making and minimizing errors [63].

There are certain limitations in our study. Although we assessed how medical graduates are provided FP training, by interviewing the faculty of department of community medicine and obstetrics and gynaecology, but we did not assess the knowledge and skills of faculty themselves regarding family planning services, which could have further provided gaps in the medical education pattern. Since this was a cross-sectional study and information was self-reported by face to face interview, there could be a chance of recall and social desirability bias. Though interviews were conducted with one respondent at one time in closed room settings, yet response bias (tendency of the participants to respond falsely to questions) cannot be ruled out [64]. This could be attributed to contamination by prior information to the participants regarding what is being asked during the interviews by the interns/nurses who had already been interviewed. So the participants might already know the correct answers before facing the actual interview, but in reality their knowledge might be less. There could be chances of selection bias due to non-recruitment of nurses from medical college in Delhi.

In-depth cause of poor knowledge and skills of participants could not be explored, and it is suggested that qualitative studies should be planned to further explore these reasons in the future. Exploring client level barriers and enablers for accessing the FP services could have further given more clarity on this issue. While the planned methodology of doing an OSCE is strong, but half of the respondents had refused to demonstrate. This aspect can be strengthened by stating it as a part of the inclusion criteria of the study, included in the consent form, and a proper explanation of what it is in future studies. The reasons for denial for practical demonstrations by medical interns and nurses can also be explored.

To conclude, our study depicted that knowledge and skills of interns and nurses regarding FP services were inadequate. The medical training pertaining to family planning services during graduation or internship, and during the job, was found to be inadequate to provide quality FP services as per guidelines of medical council of India and Government of India. Information provided through our study can be considered edifying as it attempts to provides insights into training needs, barriers and facilitators from different perspectives and hence can be considered as a baseline assessment for designing large-scale research studies and guide the policy makers in taking initiatives to strengthen preservice medical education on FP.

Based on the results of this study, it is recommended that training of interns and nurses on FP services should be given more emphasis during pre-service medical education and in-service training. Quality of in-service training of nurses including continued nursing education on FP related topics should be organized. It is recommended to improve the pre-service medical education pertaining to the FP and reproductive rights by having a consensus on core competencies in FP for medical students, identifying opportunities for building FP skills like giving case studies, conducting role plays, organzing capacity building seminars for medical students, and continued medical education for medical faculty on skill-based FP as suggested by medical council of India regarding competency and skills to be acquired by medical graduates [65]. Skill based reproductive health learning (by simulated training) should be implemented. Practical examinations can be modified by including competency skill-based assessment regarding use of contraceptive methods. Similar modifications suggested in other studies have substantiated the existing curriculum and improved the skills sets of the students [66,67]. Internship duties should include a minimum set of procedures (like IUCD insertion) and minimum number of cases/clients counseled on use of various contraceptive methods in the FP clinic/OPD. Skills and knowledge acquired during the posting might be assessed by conducting the exit tests.

There is evidence on improvement of skills and knowledge of interns on family planning and reproductive health by improved teaching curriculums. According to Berdzuli et al report 2009, United States Agency for International Development [USAID] has documented the strengthening of preservice medical education on family planning and reproductive health in Europe and Eurasia by addressing the key problems in medical and nursing schools related to FP teaching and training. The specific actions taken included implementation of the evidence based curriculum content and teaching, curricular reforms to include case based teaching and student centred learning of FP, for example use of WHO Medical Eligibility Criteria for contraceptive use and development of core competencies curriculum for the medical graduates[68]. Earlier USAID and JHIEPGO had developed National Integrated Clinical Training system to strengthen FP and reproductive preservice education and inservice training in Turkey, where it has been documented that interns FP/RH knowledge and skills especially of of IUCD insertion especially improved after this change in the training pattern[69]. Linhares et al (2015) had prepared a standardized competency matrix with list of minimum requirements that interns should be able to practice after rotatory training in the department of Obstetrics and Gynaecology [70]. Gebremeskel et al (2018) in an intervention study reported a higher proportion of interns in the intervention arm, trained under integrated FP curriculum with skill based teaching and learning, who rated being competent themselves across all contraceptive procedures as compared to the control arm (p<0.01)[66]. There are evidences from other specialities [medicine and ophthalmology] as well where competency based learning and skill assessment has resulted in better knowledge and skills of interns [71,72].

Enabling the interns and nurses to be competent in providing family planning services would also be dependent on the clients and procedure load of the clinical setting. This should be sufficient enough to meet the training needs of both medical interns and nursing students. If the clients and procedure load is not sufficient then other methods including use of simulation/models, role plays, case studies should be explored for inclusion in the teaching. Recent MCI competency based guidelines on family planning teaching has mentioned the methods of teaching as well to guide the faculty[65]. Faculty members of the medical college should take the responsibility of teaching their students with a more serious approach towards FP services to ensure universal access to sexual and reproductive health care services and rights.

## Supporting information

S1 File Study tool on assessing the knowledge and skills of the interns and nurses and training status regarding family planning methods. (DOCX)

S2 File Study tool on assessing the practices of family planning (FP) and reproductive health (RH) services in the medical colleges. (DOCX)

S3 File Hindi Version of Study tool on assessing the knowledge and skills of the interns and nurses and training status regarding family planning methods. (DOCX)

S4 File Data analysis plan regarding the categorization of responses as correct, partially correct and wrong responses. (DOCX)

S1 Table Gender based assessment of knowledge regarding various contraceptive methods in each provider category. (DOCX)

S2 Table Age wise assessment of knowledge regarding various contraceptive methods of the study participants. (DOCX)

S3 Table Assessment of knowledge regarding various contraceptive methods as per the marital status of the study participants. (DOCX)

S4 Table Teaching practices of faculty of Community Medicine and Gynaecology and Obstetrics in the medical colleges related to family planning services. (DOCX)

S5 Table Record review and observation of family planning clinics under department of obstetrics and gynaecology or community medicine in the medical colleges. (DOCX)
