# Supplementary material for: Competency assessment of the medical interns and nurses and documenting prevailing practices to provide family planning services in teaching hospitals in three states of India
Source: PLoS One. 2019 Nov 6;14(11):e0211168. doi: 10.1371/journal.pone.0211168 (PMC6834278; doi:10.1371/journal.pone.0211168)
Supplement: S1 File — (DOCX) [file pone.0211168.s001.docx]

**S1 File. Study tool on assessing the knowledge and skills of the interns and nurses and training status regarding family planning methods.**

**Title of the project: Assessment of skills of fresh medical graduates and nurses regarding family planning services**

Department of Community Medicine and School of Public Health

Post Graduate Institute of Medical Education and Research (PGIMER), Chandigarh, India

**Part A. Interview Schedule**

**Section I: Sociodemographic Variables**

1. **Unique Id. No:**
2. **Name:**
3. **Age (in completed years):**
4. **Sex:** Male: 1 Female: 2
5. **Marital Status:** Married: 1 Unmarried: 2 Separated: 3
6. **Place_____________ Roll No._____________ Phone no:_____________________**
7. **College:**
8. **Intern: 1 Nurse: 2**
9. **How long have you been working as intern or nurse?.......year……..months**

**Section II. Family planning related questions**

1. What are the various family planning methods you know of?
2. Condoms
3. IUCD
4. OCP
5. Emergency Contraceptive Pill
6. Injectable Contraceptives
7. Natural methods
8. Implantable contraceptives
9. Non hormonal non-steroidal pills
10. Permanent contraception
11. Spermicides
12. If a newly married couple / woman comes asking for contraceptives, which contraceptives would you tell them about?
13. Condoms
14. OCP
15. POP
16. IUCD
17. If a woman with one child comes asking for contraceptives, which contraceptives would you tell her about?
18. Condoms
19. OCP
20. POP
21. IUCD
22. If a woman with 3 children comes asking for contraceptives, which contraceptives would you tell her about?
    1. Condoms
    2. OCP
    3. POP
    4. IUCD
    5. Sterilisation
23. If a newly married woman (age 20 years) asks for a contraceptive, and she comes alone, without a family member, can you give her a contraceptive?
    1. Yes
    2. Yes but only after asking the family members
    3. No
24. If an unmarried woman asks for a contraceptive, and she comes alone, without a family member, can you give her a contraceptive?
    1. Yes
    2. Yes but only after asking the family members
    3. No
25. In India, is it legal to provide contraceptives to unmarried people?
    1. Yes
    2. No

**IIa. Intrauterine contraceptive devices**

1. How many types of intrauterine devices are you aware of?
   1. Copper
   2. Hormonal
   3. First generation/ Inert IUCD
2. What are the three most common conditions you will rule out before inserting Copper T?
3. Pregnancy
4. STI/HIV
5. Irregular Periods
6. Adnexal Mass/Ectopic Pregnancy
7. Multiple Sexual Partners
8. What are the most common side effects of Cu-T insertion?
9. Pain/cramps
10. Bleeding/menorrhagia/spotting/irregular bleeding
11. Infections/PID/vaginal discharge
12. Expulsion
13. Which types of CuT is available in government supply?
14. CuT 375
15. CuT 380A
16. How long Copper T 380 A offers protection?
17. 3 years
18. 5 years
19. 6-9 years
20. 10 years
21. When is Post Partum IUCD (PPIUCD) to be inserted?
22. Within 10 minutes of delivery
23. Within 48 hours
24. During Caesarean section
25. Other
26. Don’t know
27. When should consent be taken for PPIUCD insertion?
28. Antenatal period
29. Perinatal period
30. Postnatal period

**II b. Oral Contraceptive Pills (OCPs)**

1. If a woman says she is interested in using OCPs, what conditions must you rule out in her history? (At least four correct responses)
   - 1. H/o Smoking
     2. H/o Diabetes
     3. H/o Headaches
     4. H/o Cardiovascular diseases
     5. H/o Thromboembolic episodes
     6. Less than 6 weeks postpartum
     7. H/o Liver disease
     8. H/o Breast cancer
2. Can OCPs be bought over the counter?
3. Yes
4. No
5. What instructions will you give the woman who wants to use OCP?
6. When to start the pill
7. Daily intake without fail (3 weeks + 1week)
8. What to do if she misses a pill
9. Side effects

(If three answers right: correct information

If two answers right: then partial information

If one answers right: incorrect response)

1. What should a woman do if she misses 2 pills?
2. She has to **take 2 pills the next da**y
3. Again 2 pills the second next day.
4. The **couple should also use condom for 7 days**

(If three answers right: correct information

If two answers right: then partial information

If one answers right: incorrect response)

1. Can OCPs be given to:
   1. Newly married women? YES/NO
   2. Illiterate women? YES/NO
   3. Women who do not want any more children? YES/NO

(If three answers right: correct information

If two answers right: then partial information

If one answers right: incorrect response)

1. WHICH OCPs are available in the government supply?
2. Mala D
3. Mala N

**II c. Condoms**

1. What is failure rate of condoms IF USED correctly?
2. <5%
3. 6-15%
4. >15%
5. Other. Specify______________
6. Do not know
7. What are the most common two ADVANTAGES of condom?
8. Provides protection not only against pregnancy but also against STD and HIV.
9. No side effects
10. Other. Specify______________

**II d. Injectable contraceptives**

1. What kind of contraceptive is DMPA?

Medroxyprogesterone acetate. DMPA is a Progestogen-only Injectable (POI)

1. If a woman wishes to use DMPA, what questions do you need to ask her in history? (at least 2 should be mentioned)
   - 1. Pregnancy
     2. Irregular periods
     3. Breast cancer
     4. Liver disease
     5. Thromboembolic episodes (HEART ATTACK/STROKE/TIA)
2. If a woman wishes to use DMPA, what are the most important issues on which you should counsel her?
3. Menstruation related side effects
4. Delayed return of fertility
5. Don’t know
6. Is injectable contraceptive available in public health system?
7. Yes
8. No

**II. e. Post Partum Contraception**

1. What are the three prerequisites for lactational amenorrhea to be an effective contraceptive method?
2. Amenorrhea
3. Exclusive breast feeding
4. 6 months
5. Don’t know
6. A woman has delivered a healthy baby 3 months ago. She is breast feeding her baby along with top feed. Which contraceptives can be advised to her?
7. IUCD
8. Injectable
9. POP
10. Condom

**II. f. Emergency Contraception**

1. What is the type of contraception used after unprotected intercourse?
2. Emergency contraceptive pills
3. IUCD
4. Yuzpe’s method (high dose of Combined OCPs)
5. Till what time emergency contraceptive pill is effective?
6. To be consumed within 24 hours of unprotected intercourse to prevent pregnancy
7. To be consumed within 48 hours of unprotected intercourse to prevent pregnancy
8. To be consumed within 72 hours of unprotected intercourse to prevent pregnancy
9. Do not know
10. Other specify
11. A woman has used Emergency contraceptive pills 3 times in last 1 year. Now she comes to your OPD and ask for it the fourth time. Will you prescribe it again this time?
12. Yes
13. No
14. Why is centchroman (Chhaya/Saheli) different from OCPs?
    - 1. It is a non-steroidal and non-hormonal contraceptive.
      2. Don’t know
15. How frequently should centchroman be taken?
    - 1. 1 tablet weekly
      2. Don’t know

**II g. Tubectomy**

1. Have you ever seen the eligibility checklist for Tubectomy?
2. Yes
3. No
4. Have you seen

2a Tubectomy operation

1. Yes
2. No

2b consent form for Tubectomy

1. Yes
2. No

**II h Natural family planning methods**

1. Which natural family planning methods have you heard of?
2. Rhythm method/safe period/calendar
3. Lactational Amenorrhea
4. Basal body temperature
5. Cervical mucus
6. Abstinence
7. Coitus interruptus

**Section III. Trainings on FP**

1. **Status of training received by interns on family planning methods.**
2. Have you ever attended any class on family planning methods in your MBBS period?
3. Yes
4. No
5. Were you posted in family planning clinic during internship/MBBS?
   1. Yes
   2. No

If Yes

1. For how many days were you posted in family planning clinic during internship/MBBS?
2. In which department were you posted?
3. Community Medicine - ______ DAYS
4. Gynecology and obstetrics - ______ DAYS
5. Have you ever observed the insertion of the IUD?
6. Yes
7. No
8. Have you ever inserted an IUCD yourself
9. Yes
10. No
11. Have you ever inserted an IUCD on a dummy/model?
12. Yes
13. No
14. Have you ever seen an IUCD removal?
    1. Yes
    2. No
15. Have you ever removed an IUCD yourself?
16. Yes
17. No
18. Have you ever observed counselling on different family planning methods?
    1. Yes
    2. No
19. Have you ever seen the following
20. Condom
21. OCP
22. IUCD
23. DMPA
24. ECP
25. Centchroman
26. Hormonal IUD
27. Dermal implants
28. Spermicides
29. Have you ever seen MEC Wheel (Medical Eligibility Criteria Wheel)?
30. Yes
31. No
32. Have you ever seen anyone using MEC wheel on a patient (medical eligibility criteria wheel)?
33. Yes
34. No
35. **Status of training received by nurses on family planning methods.**
36. Have you ever attended any class on family planning methods in your training period?
37. Yes
38. No
39. Were you posted in family planning clinic during training?
    1. Yes
    2. No

If Yes

1. For how many days were you posted in family planning clinic during your training period?
2. Have you ever observed the insertion of the IUD?
3. Yes
4. No
5. Have you ever inserted an IUCD yourself
6. Yes
7. No
8. Have you ever inserted an IUCD on a dummy/model?
9. Yes
10. No
11. Have you ever seen an IUCD removal?
    1. Yes
    2. No
12. Have you ever removed an IUCD yourself?
13. Yes
14. No
15. Have you ever observed counselling on different family planning methods?
    1. Yes
    2. No
16. Have you ever seen the following
17. Condom
18. OCP
19. IUCD
20. DMPA
21. ECP
22. Centchroman
23. Hormonal IUD
24. Spermicides
25. Dermal implants
26. Have you ever seen MEC Wheel (Medical Eligibility Criteria Wheel)?
27. Yes
28. No
29. Have you ever seen anyone using MEC wheel on a patient (medical eligibility criteria wheel)?
30. Yes
31. No

**PART B. Observation check list as per Objective Structured Clinical Examination (OSCE)**

**B1. Insertion of Cu-T 380A**

**Observation:** Observe if the participant is performing the following steps of insertion of Cu-T 380 A in their correct sequence (as necessary) and technique.

If participant does not know how to perform/ refuses to perform – Score 0

| SN | TASK | Score 0/1/2 | Remarks |  |
| --- | --- | --- | --- | --- |
|  |  |  |  |  |
|  |  |  |  |  |
| 1 | Washes hands and wear gloves | 1 |  |  |
|  |  |  |  |  |
| 2 | Insert the sterile sound using the “no touch” technique | 1 |  |  |
|  |  |  |  |  |
| 3 | Load the IUCD in its sterile package | 2 |  |  |
|  |  |  |  |  |
| 4 | Set the blue depth-gauge to the measurement of the uterus | 1 |  |  |
|  |  |  |  |  |
| 5 | Carefully insert the loaded IUCD, and release it into the uterus using the “withdrawal” technique | 2 |  |  |
|  |  |  |  |  |
| 6 | Take out the plunger. | 1 |  |  |
|  |  |  |  |  |
|  |  |  |  |  |
| 7 | Partially withdraw the insertion tube until the IUCD strings can be seen. | 1 |  |  |
|  |  |  |  |  |
| 8 | Use sterile scissors to cut the IUCD strings  to 3-4 cm length in the vagina | 1 |  |  |
|  |  |  |  |  |

Pass Score = 5/10 Student Score = _________

Pass- Yes No

**B2.** **Demonstration on use of condom**

If participant does not know how to perform/ refuses to perform – Score 0

Score “1” for each point conducted correctly or mark “0” if the task is not done or incorrectly done and calculate the Score.

| S.N | TASK | Score |
| --- | --- | --- |
| 1 | Check the expiry date on the wrapper |  |
| 2 | Open the package without tearing the condom |  |
| 3 | Do not use teeth to open the wrapper |  |
| 4 | Hold the condom by the last ½ inch at the tip, making sure  to squeeze out any air |  |
| 5 | Put the condom on the tip of the thumb. |  |
| 6 | While still pinching the tip, unroll the condom down the shaft  to the base of the thumb. |  |
| 7 | Remove the condom by rolling it off. |  |
| 8 | Throw the condom in the dustbin. |  |

Pass Score = 5/8

Student Score = _______

Pass- Yes No

**B3. Observation Regarding the use of a Medical Eligibility Criteria Wheel**

If participant does not know how to perform/ refuses to perform – Score 0

If a 25 year old woman, with 2 children, youngest being 1 year old, comes asking for DMPA. Her medical condition shows:

1. No breast lump,
2. No h/o heart attack, jaundice, diabetes.
3. She has normal periods, last menstrual period was 6 days ago.

Use a Medical eligibility criteria wheel, and tell her whether she can use DMPA.

Pass- Yes No

**Part C. Interview of faculty regarding FP teaching and observation of training facilities**

1. Department: Obstetrics and gynecology/ Community medicine
2. Where do students receive Family planning practical training?
   - 1. Family planning room
     2. Skill lab
     3. Other
3. What facilities are available to train students on FP?
4. Dummy/ model/ patients
5. Samples of contraceptives:
6. Copper IUD
7. Hormonal IUD
8. Combined Oral pill
9. POP
10. DMPA
11. Condoms
12. Implant
13. Spermicides
14. Emergency Contraceptive Pill
15. What Training Modules are available for Family Planning practical training
16. Textbook
17. GOI guidelines, IF YES; see for the availability
18. If Mannequins are available, what type?
19. Is MEC wheel available?
20. When was the last student visit conducted?
21. Do you only demonstrate Family planning methods to them or also allow them to practice hands on?
