# Supplementary material for: Competency assessment of the medical interns and nurses and documenting prevailing practices to provide family planning services in teaching hospitals in three states of India
Source: PLoS One. 2019 Nov 6;14(11):e0211168. doi: 10.1371/journal.pone.0211168 (PMC6834278; doi:10.1371/journal.pone.0211168)
Supplement: S2 File — (DOCX) [file pone.0211168.s002.docx]

**S2 File. Study tool on assessing the practices of family planning (FP) and reproductive health (RH) services in the medical colleges.**

**Title of the project: Assessment of skills of fresh medical graduates and nurses regarding family planning services**

Department of Community Medicine and School of Public Health

Post Graduate Institute of Medical Education and Research (PGIMER), Chandigarh, India

1. Date of observation:
2. Facility:
3. Name of observer:

**Part 1: Observation of OPD room and waiting area**

| QN | Question | Response |
| --- | --- | --- |
| 1 | While in waiting area, can other patients see patients in the OPD who are having consultation with doctor? | Yes/ No |
| 2 | Is there auditory privacy in the OPD (while in waiting area, can you/ other patients hear the conversations between patients who are having consultation with doctor/ nurse)? | Yes/ No |
| 4 | Is the door of OPD room shut? | - The door of OPD room is shut =1 - The door is not shut but a curtain is drawn, so patient talking to doctor cannot be seen =2 - The door is open and there is no curtain =3 |

**Part 2: Records review**

| 1 | Number of persons who provided following family planning services: | Number of persons provided this service in last month | Number of pieces distributed |
| --- | --- | --- | --- |
|  | - IUDs (interval) |  | NA |
|  | - IUD (postpartum) |  | NA |
|  | - Female sterilization |  | NA |
|  | - Male sterilization |  | NA |
|  | - Injectable contraceptive (DMPA) |  | NA |
|  | - Combined Oral pills |  |  |
|  | - Condoms |  |  |
|  | - Centchroman |  |  |
|  | - ECs |  |  |

**Part 3: Stocks position** (last 1 month)

| 3 | Supplies of following available in dispensary / store? | Y / N | If no, for how long is this item not available? |
| --- | --- | --- | --- |
|  | - Pregnancy tests |  |  |
|  | - Oral pills |  |  |
|  | - Condoms |  |  |
|  | - Injectables |  |  |
|  | - ECs |  |  |
|  | - CopperT 380A |  |  |
|  | - Copper T 375 |  |  |
| 4 | Is FP-LMIS functional here? | Yes  No  Yes, but there are problems |  |

**Part 4: Interview of faculty/medical officer in charge of FP clinics regarding practices related to family planning and reproductive health services**

You have been providing services at this facility. Several women come here for receiving SRH and family planning services also. I wish to understand from you about facilities and barriers that you face in strengthening SRH services at this hospital.

| SN | Question | Response | Code |
| --- | --- | --- | --- |
| 8 | How is the eligibility of woman assessed for PPIUCD? | Using checklist  Verbal questioning |  |
| 9 | Does the department have a medical eligibility criteria (MEC) wheel? | Y  N |  |
| 10 | If yes, Is it available in sufficient numbers and is used in OPD for assessment of eligibility for FP? |  |  |
| 11 | If no, do you use any other medical eligibility criteria check list before advising about contraceptive methods? |  |  |

***Thank the provider and close the interview***
