# Supplementary material for: Competency assessment of the medical interns and nurses and documenting prevailing practices to provide family planning services in teaching hospitals in three states of India
Source: PLoS One. 2019 Nov 6;14(11):e0211168. doi: 10.1371/journal.pone.0211168 (PMC6834278; doi:10.1371/journal.pone.0211168)
Supplement: S3 File — (DOCX) [file pone.0211168.s003.docx]

**S3 File: Hindi Version of Study tool on assessing the knowledge and skills of the interns and nurses and training status regarding family planning methods.**

**Title of the project: Strengthening Evidence-based Family Planning Service in Pre-service Medical Education**

*Department of Community Medicine, School of Public Health*

*Post Graduate Institute of Medical Education and Research (PGIMER), Chandigarh, India*

भाग ए साक्षात्कार अनुसूची

**धारा I: समाजशास्त्र वैरिएबल**

1. अद्वितीय आईडी। न:

2. नाम:

3. आयु (पूर्ण वर्षों में):

4. लिंग: पुरुष: 1 महिला: 2

5. वैवाहिक स्थिति: विवाहित: 1 अविवाहित: 2 अलग: 3

6. प्लेस_____________ रोल नंबर ._____________ फोन नंबर: _____________________

7. कॉलेज:

8. आंतरिक/इंटर्न: 1 नर्स: 2

9. आप कितने समय से इंटर्न या नर्स के रूप में काम कर रहे हैं? ....... साल ...... .. माह

धारा II परिवार नियोजन संबंधित प्रश्न

10. आप जानते हैं कि विभिन्न परिवार नियोजन विधियों क्या हैं?

1. कंडोम
2. IUCD
3. ओसीपी
4. आपातकालीन गर्भनिरोधक पिल्ला
5. इंजेक्शन योग्य गर्भनिरोधक
6. प्राकृतिक तरीकों
7. प्रत्यारोपण गर्भ निरोधक
8. गैर हार्मोनल गैर-स्टेरॉयड गोलियां
9. स्थायी गर्भनिरोधक
10. शुक्राणुनाशक

11. अगर एक नव विवाहित जोड़े / महिला गर्भ निरोधकों के लिए पूछती है, तो आप किस गर्भ निरोधकों के बारे में बताएंगे?

1. कंडोम
2. ओसीपी
3. पॉप
4. IUCD

12. अगर एक बच्चा वाली महिला गर्भ निरोधकों के लिए पूछती है, तो आप उसे किस गर्भ निरोधक के बारे में बताएंगे?

1. कंडोम
2. ओसीपी
3. पॉप
4. IUCD

13. अगर 3 बच्चे वाली एक महिला गर्भ निरोधकों के लिए पूछती है, तो आप उसे किस गर्भ निरोधक के बारे में बताएंगे?

1. कंडोम
2. ओसीपी
3. पॉप
4. IUCD
5. बंध्याकरण

14. अगर एक नव विवाहित महिला (20 साल की उम्र) गर्भनिरोधक के लिए पूछती है, और वह परिवार के सदस्य के बिना अकेली आती है, तो क्या आप उसे गर्भनिरोधक दे सकते हैं?

1. हाँ
2. हाँ, लेकिन परिवार के सदस्यों से पूछने के बाद ही
3. नहीं

15. अगर अनौपचारिक महिला गर्भनिरोधक के लिए पूछती है, और वह परिवार के सदस्य के बिना अकेली आती है, तो क्या आप उसे गर्भनिरोधक दे सकते हैं?

1. हाँ
2. हाँ, लेकिन परिवार के सदस्यों से पूछने के बाद ही
3. नहीं

16. भारत में, अविवाहित लोगों को गर्भ निरोधकों को प्रदान करना कानूनी है?

1. हाँ
2. नहीं
3. **इंट्राउटरिन डिवाइस**

17. आप कितने प्रकार के इंट्रायूटरिन डिवाइस के बारे में जानते हैं?

1. तांबा
2. हार्मोनल
3. पहली पीढ़ी / निष्क्रिय आईयूसीडी

18. कॉपर टी डालने से पहले आप तीन सबसे आम स्थितियों को किस प्रकार निषेध करेंगे?

1. गर्भावस्था
2. एसटीआई / एचआईवी
3. अनियमित काल
4. एडनेक्सल मास / एक्टोपिक गर्भावस्था
5. एकाधिक यौन साथी

19. क्यू-टिनरशन के सबसे आम दुष्प्रभाव क्या हैं?

1. दर्द / ऐंठन
2. रक्तस्राव / मनोहरिया / स्पॉटिंग / अनियमित रक्तस्राव
3. संक्रमण / पीआईडी ​​/ योनि निर्वहन
4. निष्कासन

20. सरकारी आपूर्ति में कौन सी प्रकार सीयूटी उपलब्ध है?

1. सीयूटी 375
2. क्यूटी 380 ए

21. कॉपर टी 380 ऑफर्स सुरक्षा कब तक?

1. 3 साल
2. 5 वर्ष
3. 6-9 साल
4. 10 साल

22. पोस्ट पार्टम आईयूसीडी (पीपीआईयूसीडी) कब सम्मिलित किया जाना है?

1. प्रसव के 10 मिनट के भीतर
2. 48 घंटों के भीतर
3. Caesarean अनुभाग के दौरान
4. अन्य
5. पता नहीं

23. पीपीआईयूसीडी सम्मिलन के लिए सहमति कब लेनी चाहिए?

1. प्रसव की अवधि
2. प्रसव की अवधि
3. प्रसवोत्तर काल
4. **मौखिक गर्भनिरोधक गोलियाँ (ओसीपी)**

24. अगर कोई महिला कहती है कि वह ओसीपी का उपयोग करने में रूचि रखती है, तो आप अपने इतिहास में किस शर्त से इंकार कर सकते हैं? (कम से कम चार सही प्रतिक्रियाएं)

1. एच / ओ धूम्रपान
2. एच / ओ मधुमेह
3. एच / ओ सिरदर्द
4. एच / ओ कार्डियोवैस्कुलर बीमारियां
5. एच / ओ थ्रोम्बोम्बोलिक एपिसोड
6. 6 सप्ताह से कम postpartum
7. एच / ओ जिगर की बीमारी
8. एच / ओ स्तन कैंसर

25. काउंटर पर ओसीपी खरीदा जा सकता है?

1. हाँ
2. नहीं

26. आप उस महिला को कौन से निर्देश देंगे जो ओसीपी का उपयोग करना चाहता है?

1. गोली कब शुरू करें
2. असफल बिना दैनिक सेवन (3 सप्ताह + 1week)
3. अगर वह गोली मारती है तो क्या करें
4. दुष्प्रभाव

(यदि तीन सही सही हैं: सही जानकारी

यदि दो सही जवाब देते हैं: तो आंशिक जानकारी

अगर कोई सही जवाब देता है: गलत प्रतिक्रिया)

27. अगर वह 2 गोलियों को याद करती है तो एक औरत को क्या करना चाहिए?

1. उसे अगले दिन 2 गोलियां लेनी होंगी
2. दूसरे दिन फिर से 2 गोलियाँ।
3. जोड़े को 7 दिनों के लिए कंडोम का भी उपयोग करना चाहिए

(यदि तीन सही सही हैं: सही जानकारी

यदि दो सही जवाब देते हैं: तो आंशिक जानकारी

अगर कोई सही जवाब देता है: गलत प्रतिक्रिया)

28. ओसीपी को दिया जा सकता है:

1. नई शादीशुदा महिलाएं? हाॅं नही
2. निरक्षर महिलाएं? हाॅं नही
3. महिलाएं जो अब और बच्चों को नहीं चाहती हैं? हाॅं नही

(यदि तीन सही सही हैं: सही जानकारी

यदि दो सही जवाब देते हैं: तो आंशिक जानकारी

अगर कोई सही जवाब देता है: गलत प्रतिक्रिया)

2 9। सरकारी आपूर्ति में कौन से ओसीपी उपलब्ध हैं?

1. माला डी
2. माला एन

**द्वितीय सी; कंडोम**

30. कंडोम की विफलता दर क्या है अगर सही ढंग से उपयोग किया जाता है?

1. <5%
2. 6-15%
3. 15%
4. अन्य। उल्लिखित करना______________
5. पता नहीं

31. कंडोम के सबसे आम दो फायदे क्या हैं?

1. न केवल गर्भावस्था के खिलाफ बल्कि एसटीडी और एचआईवी के खिलाफ भी सुरक्षा प्रदान करता है।
2. कोई दुष्प्रभाव नहीं
3. अन्य। उल्लिखित करना______________

**द्वितीय डी; इंजेक्शन योग्य गर्भ निरोधक**

32. डीएमपीए किस तरह का गर्भ निरोधक है?

1. Medroxyprogesterone एसीटेट।
2. डीएमपीए एक प्रोजेस्टोजेन-इंजेक्शन योग्य (पीओआई) है

33. यदि कोई महिला डीएमपीए का उपयोग करना चाहती है, तो उसे इतिहास में उससे पूछने के लिए आपको किन प्रश्नों की आवश्यकता है? (कम से कम 2 का उल्लेख किया जाना चाहिए)

1. गर्भावस्था
2. अनियमित अवधि
3. स्तन कैंसर
4. जिगर की बीमारी
5. थ्रोम्बोम्बोलिक एपिसोड (हृदय अटैक / स्ट्रोक / टीआईए)

34. यदि कोई महिला डीएमपीए का उपयोग करना चाहती है, तो सबसे महत्वपूर्ण मुद्दे क्या हैं जिन पर आपको सलाह देनी चाहिए?

1. मासिक धर्म से संबंधित दुष्प्रभाव
2. प्रजनन क्षमता में देरी की वापसी
3. पता नहीं

35. सार्वजनिक स्वास्थ्य प्रणाली में इंजेक्शन योग्य गर्भनिरोधक उपलब्ध है?

1. हाँ
2. नहीं

**द्वितीय। ई। पोस्ट पार्टम गर्भनिरोधक**

36. लैक्टेशनल अमेनोरिया के लिए एक प्रभावी गर्भ निरोधक विधि होने के लिए तीन आवश्यकताएं क्या हैं?

amenorrhea

1. विशेष स्तनपान
2. 6 महीने
3. पता नहीं

37. एक महिला ने 3 महीने पहले एक स्वस्थ बच्चा दिया है। वह शीर्ष फ़ीड के साथ अपने बच्चे को स्तनपान कर रही है। उसे किस गर्भ निरोधकों की सलाह दी जा सकती है?

1. IUCD
2. इंजेक्शन
3. पॉप
4. कंडोम

**द्वितीय। च। आपातकालीन गर्भनिरोधक**

38. असुरक्षित संभोग के बाद गर्भनिरोधक के प्रकार का क्या उपयोग किया जाता है?

1. आपातकालीन गर्भ निरोधक गोलियाँ
2. IUCD
3. युजपे की विधि (संयुक्त ओसीपी की उच्च खुराक)

39. जब तक आपातकालीन गर्भनिरोधक गोली प्रभावी होती है?

1. गर्भावस्था को रोकने के लिए असुरक्षित संभोग के 24 घंटों के भीतर उपभोग किया जाना चाहिए
2. गर्भावस्था को रोकने के लिए असुरक्षित संभोग के 48 घंटों के भीतर उपभोग किया जाना चाहिए
3. गर्भावस्था को रोकने के लिए असुरक्षित संभोग के 72 घंटों के भीतर उपभोग किया जाना चाहिए
4. मालूम नहीं
5. अन्य निर्दिष्ट करें

40. एक महिला ने पिछले 1 साल में आपातकालीन गर्भनिरोधक गोलियां 3 बार उपयोग की हैं। अब वह आपके ओपीडी में आती है और चौथे बार पूछती है। क्या आप इस बार फिर से लिखेंगे?

1. हाँ
2. नहीं

41. केंद्र सरकार (छाया / साहेली) ओसीपी से अलग क्यों है?

1. यह एक गैर-स्टेरॉयड और गैर-हार्मोनल गर्भ निरोधक है।
2. पता नहीं

42. सेंट्र्रोमन को कितनी बार ले जाना चाहिए?

1. 1 टैबलेट साप्ताहिक
2. पता नहीं

द्वितीय जी महिला नसबंदी

43. क्या आपने कभी ट्यूबेटोमी के लिए पात्रता चेकलिस्ट देखी है?

1. हाँ
2. नहीं

44. क्या आपने देखा है

2 ए ट्यूबेटोमी ऑपरेशन

1. हाँ
2. नहीं

Tubectomy के लिए 2 बी सहमति फॉर्म

1. हाँ
2. नहीं

द्वितीय मानव परिवार नियोजन विधियों

45. आपने किस प्राकृतिक परिवार नियोजन विधियों के बारे में सुना है?

1. ताल विधि / सुरक्षित अवधि / कैलेंडर
2. लैक्टेशनल अमेनोरेरिया
3. बुनियादी दैहिक तापमान
4. ग्रीवा बलगम
5. संयम
6. कोइटस इंटरप्टस

धारा III

ए) परिवार नियोजन पद्धतियों पर प्रशिक्षित प्रशिक्षण - इंटर्न

46. ​​क्या आपने कभी अपनी एमबीबीएस अवधि में परिवार नियोजन विधियों पर किसी भी कक्षा में भाग लिया है?

1. हाँ
2. नहीं

47. क्या आप इंटर्नशिप / एमबीबीएस के दौरान परिवार नियोजन क्लिनिक में तैनात थे?

1. मैं। हाँ
2. नहीं

अगर हाँ

48. इंटर्नशिप / एमबीबीएस के दौरान परिवार नियोजन क्लिनिक में आपने कितने दिन काम किए हैं?

49. आपने किस विभाग में पोस्ट किया था?

1. सामुदायिक चिकित्सा - ______ दिन
2. स्त्री रोग और प्रसूति - ______ दिन

50. क्या आपने कभी आईयूडी के सम्मिलन को देखा है?

1. हाँ
2. नहीं

51. क्या आपने कभी एक आईयूसीडी डाली है

1. हाँ
2. नहीं

52. क्या आपने कभी एक डमी / मॉडल पर आईयूसीडी डाली है?

1. हाँ
2. नहीं

53. क्या आपने कभी आईयूसीडी हटाने को देखा है?

1. हाँ
2. नहीं

54. क्या आपने कभी आईयूसीडी को हटा दिया है?

1. हाँ
2. नहीं

55. क्या आपने कभी विभिन्न परिवार नियोजन विधियों पर परामर्श देखा है?

1. हाँ
2. नहीं

56. क्या आपने कभी निम्नलिखित देखा है

1. कंडोम
2. ओसीपी
3. IUCD
4. DMPA
5. ECP
6. Centchroman
7. हार्मोनल आईयूडी
8. त्वचीय प्रत्यारोपण
9. शुक्राणुनाशकों

57. क्या आपने कभी एमईसी व्हील (मेडिकल पात्रता मानदंड व्हील) देखा है?

1. हाँ
2. नहीं

58. क्या आपने कभी किसी मरीज़ (मेडिकल पात्रता मानदंड पहिया) पर एमईसी व्हील का उपयोग करके देखा है?

1. हाँ
2. नहीं

बी) परिवार नियोजन पद्धतियों पर प्रशिक्षण प्राप्त - एएनएम / जीएनएम

59. क्या आपने कभी अपनी प्रशिक्षण अवधि में परिवार नियोजन विधियों पर किसी भी कक्षा में भाग लिया है?

1. हाँ
2. नहीं

60. क्या आप प्रशिक्षण के दौरान परिवार नियोजन क्लिनिक में तैनात थे?

1. हाँ
2. नहीं

अगर हाँ

61. आपके प्रशिक्षण अवधि के दौरान परिवार नियोजन क्लिनिक में आपने कितने दिन पोस्ट किए थे?

62. क्या आपने कभी आईयूडी के सम्मिलन को देखा है?

1. हाँ
2. नहीं

63. क्या आपने कभी एक आईयूसीडी डाली है

1. हाँ
2. नहीं

64. क्या आपने कभी डमी / मॉडल पर आईयूसीडी डाली है?

1. हाँ
2. नहीं

65. क्या आपने कभी आईयूसीडी हटाने को देखा है?

1. हाँ
2. नहीं

66. क्या आपने कभी आईयूसीडी को हटा दिया है?

1. हाँ
2. नहीं

67. क्या आपने कभी विभिन्न परिवार नियोजन विधियों पर परामर्श देखा है?

1. हाँ
2. नहीं

68. क्या आपने कभी निम्नलिखित देखा है

1. कंडोम
2. ओसीपी
3. IUCD
4. DMPA
5. ECP
6. Centchroman
7. हार्मोनल आईयूडी
8. शुक्राणुनाशकों
9. त्वचीय प्रत्यारोपण

69. क्या आपने कभी एमईसी व्हील (मेडिकल पात्रता मानदंड व्हील) देखा है?

1. हाँ
2. नहीं

70. क्या आपने कभी किसी मरीज़ (मेडिकल पात्रता मानदंड पहिया) पर एमईसी व्हील का उपयोग करके देखा है?

1. हाँ
2. नहीं

उद्देश्य बी संरचित नैदानिक ​​परीक्षा (ओएससीई) के अनुसार भाग बी निरीक्षण जांच सूची

सी -1। क्यू-टी 380 ए का सम्मिलन

निरीक्षण: निरीक्षण करें कि प्रतिभागी अपने सही अनुक्रम (आवश्यकतानुसार) और तकनीक में क्यू-टी 380 ए के सम्मिलन के निम्नलिखित चरणों का पालन कर रहा है या नहीं।

यदि प्रतिभागी को यह नहीं पता कि प्रदर्शन करने / प्रदर्शन करने से इंकार कैसे किया जाता है - स्कोर 0

एसनो टास्क स्कोर 0/1/2 टिप्पणियां

1 हाथ धोना और दस्ताने पहनना 1

2 "नो टच" तकनीक 1 का उपयोग करके बाँझ ध्वनि डालें

3 आईयूसीडी को अपने बाँझ पैकेज 2 में लोड करें

4 गर्भाशय 1 के माप के लिए नीली गहराई-गेज सेट करें

5 लोड किए गए IUCD को ध्यान से सम्मिलित करें, और इसे "वापसी" तकनीक 2 को गर्भाशय में छोड़ दें

6 प्लंबर को बाहर निकालो। 1

7 आईयूसीडी तारों को देखे जाने तक आंशिक रूप से प्रविष्टि ट्यूब को वापस ले लें। 1

8 योनि 1 में आईयूसीडी तारों को 3-4 सेमी लंबाई में कटौती करने के लिए बाँझ कैंची का उपयोग करें

पास स्कोर = 5/10 छात्र स्कोर = _________

पास- हां नहीं

**सी-2. कंडोम के उपयोग पर प्रदर्शन**

यदि प्रतिभागी को यह नहीं पता कि प्रदर्शन करने / प्रदर्शन करने से इंकार कैसे किया जाता है - स्कोर 0

प्रत्येक बिंदु के लिए स्कोर "1" सही ढंग से आयोजित किया गया है या "0" चिह्नित करें यदि कार्य नहीं किया गया है या गलत तरीके से किया गया है और स्कोर की गणना करें।

S.no टास्क स्कोर

1 रैपर पर समाप्ति तिथि की जांच करें

2 कंडोम फाड़ने के बिना पैकेज खोलें

3 रैपर खोलने के लिए दांतों का उपयोग न करें

4 टिप पर अंतिम ½ इंच तक कंडोम पकड़ो, किसी भी हवा को निचोड़ने के लिए सुनिश्चित करें

5 अंगूठे की नोक पर कंडोम रखो।

6 अभी भी टिप को पिंच करते समय, कंडोम को अंगूठे के नीचे शाफ्ट के नीचे अनलोल करें

7 इसे घुमाकर कंडोम को हटा दें।

8 कंडोम को कचरे में फेंक दो।

पास स्कोर = 5/8

छात्र स्कोर = _______

पास- हां नहीं

**सी 3। मेडिकल पात्रता मानदंड व्हील के उपयोग के संबंध में निरीक्षण**

यदि प्रतिभागी को यह नहीं पता कि प्रदर्शन करने / प्रदर्शन करने से इंकार कैसे किया जाता है - स्कोर 0

यदि 25 वर्षीय महिला, 2 बच्चों के साथ, 1 वर्ष की उम्र में सबसे छोटी, डीएमपीए मांग रही है। उसकी चिकित्सा स्थिति से पता चलता है:

1. कोई स्तन गांठ नहीं,
2. कोई एच / ओ दिल का दौरा, पीलिया, मधुमेह नहीं।
3. उसकी सामान्य अवधि है, पिछले मासिक धर्म की अवधि 6 दिन पहले थी।
4. एक मेडिकल पात्रता मानदंड पहिया का उपयोग करें, और उसे बताएं कि क्या वह डीएमपीए का उपयोग कर सकती है।

पास- हां नहीं

**सी-4। प्रशिक्षण सुविधाओं का निरीक्षण:**

Obstetrics और स्त्री रोग / समुदाय दवा

1. छात्रों को परिवार नियोजन व्यावहारिक प्रशिक्षण कहां मिलता है?

1. परिवार नियोजन कक्ष
2. कौशल प्रयोगशाला
3. अन्य

2. एफपी पर छात्रों को प्रशिक्षित करने के लिए कौन सी सुविधाएं उपलब्ध हैं?

1. डमी / मॉडल / रोगी
2. गर्भ निरोधकों के नमूने:
3. कॉपर आईयूडी
4. हार्मोनल आईयूडी
5. संयुक्त मौखिक गोली
6. पॉप
7. DMPA
8. कंडोम
9. प्रत्यारोपण
10. शुक्राणुनाशकों
11. झ। आपातकालीन गर्भनिरोधक पिल्ला

3. परिवार नियोजन व्यावहारिक प्रशिक्षण के लिए कौन सी प्रशिक्षण मॉड्यूलर उपलब्ध है

पाठयपुस्तक

भारत सरकार के दिशानिर्देश, यदि हां; उपलब्धता के लिए देखें

4. यदि मैनचेक्विन उपलब्ध हैं, तो किस प्रकार का?

5. क्या एमईसी व्हील उपलब्ध है?

6. आखिरी स्टुडेंटविसिट कब आयोजित किया गया था?

7. क्या आप केवल उनके लिए परिवार नियोजन विधियों का प्रदर्शन करते हैं या उन्हें हैंडसन का अभ्यास करने की अनुमति भी देते हैं?
