# Supplementary material for: Competency assessment of the medical interns and nurses and documenting prevailing practices to provide family planning services in teaching hospitals in three states of India
Source: PLoS One. 2019 Nov 6;14(11):e0211168. doi: 10.1371/journal.pone.0211168 (PMC6834278; doi:10.1371/journal.pone.0211168)
Supplement: S4 File — (DOCX) [file pone.0211168.s004.docx]

**S4 File. Data analysis plan regarding the categorization of responses as correct, partially correct and wrong responses.**

| Question | Answers | Response |  |  |  |  |
| --- | --- | --- | --- | --- | --- | --- |
|  |  |  | Complete/Correct information Code 2 | Partial information  Code 1 | Incomplete/Incorrect information  Code 0 | Don’t know  Code 9 |
| If a newly married couple / woman comes asking for contraceptives, which contraceptives would you tell them about? | 1. Condoms 2. OCP 3. POP 4. IUCD | 1-4  Also combination  Like 1-2,1-3,1-4, 2-3,…. 1-2-3-4. | Any >2/4 of i,ii,iii,iv options | Any 2/4 of i,ii,iii,iv options | 1/4 of i,ii,iii,iv options | -- |
| If a woman with one child comes asking for contraceptives, which contraceptives would you tell her about? | 1. Condoms 2. OCP 3. POP 4. IUCD | 1-4  Also combination  Like 1-2,1-3,1-4, 2-3,…. 1-2-3-4. | Any >2/4 of i,ii,iii,iv options | Any 2/4 of i,ii,iii,iv options | 1/4 of i,ii,iii,iv options | -- |
| If a woman with 3 children comes asking for contraceptives, which contraceptives would you tell her about? | - 1. Condoms   2. OCP   3. POP   4. IUCD   5. Sterilization | 1-4  Also combination  Like 1-2,1-3,1-4 1-5, 2-3,…. 1-2-3-4-5. | Any >2/5 of i,ii,iii,iv,v options | Any 2/5 of i,ii,iii,iv,v options | Any 1/5 of i,ii,iii,iv,v options | -- |
| If a newly married woman (age 20 years) asks for a contraceptive, and she comes alone, without a family member, can you give her a contraceptive? | - 1. Yes   2. Yes but only after asking the family members   3. No | 1 or 2 or 3 or 9 | Only option i | N/A | Option ii or iii | -- |
| If an unmarried woman asks for a contraceptive, and she comes alone, without a family member, can you give her a contraceptive? | - 1. Yes   2. Yes but only after asking the family members   3. No | 1 or 2 or 3 or 9 | Only option i | N/A | Option ii or iii | -- |
| In India, is it legal to provide contraceptives to unmarried people? | - 1. Yes   2. No | 1 or 2 or 9 | Only option i | N/A | Option ii | -- |
| How many types of intrauterine devices are you aware of? | - 1. Copper   2. Hormonal   3. First generation/ Inert IUCD | 1 or 2 or 3 or 9  Combinations 1-2, 1-3, ….. 1-2-3 | Any 2/3 of i,ii,iii options | Any 1/3 of i,ii,iii options | O/3 of i,ii,iii options | -- |
| What are the three most common conditions you will rule out before inserting Copper T? | 1. Pregnancy 2. STI/HIV 3. Irregular Periods 4. Adnexal Mass/Ectopic Pregnancy 5. Multiple Sexual Partners | 1-4 | Any >2/5 of i,ii,iii,iv,v options | Any 2/5 of i,ii,iii,iv,v options | Any 1/5 of i,ii,iii,iv,v options | -- |
| What are the most common side effects of Cu-T insertion? | 1. Pain/cramps 2. Bleeding/menorrhagia/spotting/irregular bleeding 3. Infections/PID/vaginal discharge 4. Expulsion | 1-5 | Any >2/4 of i,ii,iii,iv options | Any 2/4 of i,ii,iii,iv options | Any 1/4 of i,ii,iii,iv options | -- |
| Which types of CuT is available in government supply? | 1. CuT 375 2. CuT 380A | 1-2 or both | Both 2/2 of i,ii options | Any 1/2 i,ii option | 0/2 of i,ii option | -- |
| How long Copper T 380 A offers protection? | 1. 3 years 2. 5 years 3. 6-9 years 4. 10 years | 1-4 | Only option iv | N/A | Other option than option iv | -- |
| When is Post Partum IUCD (PPIUCD) to be inserted? | 1. Within 10 minutes of delivery 2. Within 48 hours 3. During Caesarean section 4. Other 5. Don’t know | 1-4 | Any >2/4 of i,ii,iii,iv options | Any 2/4 of i,ii,iii,iv options | Any 1/4 of i,ii,iii,iv options | Option v |
| When should consent be taken for PPIUCD insertion? | 1. Antenatal period 2. Perinatal period 3. Postnatal period | 1-3 or all | Any ≥2/3 of i,ii,iii options | Any 1/3 of i,ii,iii options | 0/3 of i,ii,iii options | -- |
| If a woman says she is interested in using OCPs, what conditions must you rule out in her history? (At least four correct responses) | - - 1. H/o Smoking     2. H/o Diabetes     3. H/o Headaches     4. H/o Cardiovascular diseases     5. H/o Thromboembolic episodes     6. Less than 6 weeks postpartum     7. H/o Liver disease     8. H/o Breast cancer | 1-8 | Any ≥4/8 of i,ii,iii,iv,v,vi,vii,viii options | Any 3/8 of i,ii,iii,iv,v,vi,vii,viii options | Any <3/8 of i,ii,iii,iv,v,vi,vii,viii options | -- |
| Can OCPs be bought over the counter? | 1. Yes 2. No | 1 or 2 | Option i | N/A | Option ii | -- |
| What instructions will you give the woman who wants to use OCP? | 1. When to start the pill 2. Daily intake without fail (3 weeks + 1week) 3. What to do if she misses a pill 4. Side effects | 1-4 | Any ≥3/4 of i,ii,iii,iv options | Any 2/4 of i,ii,iii,iv options | Any 1/4 of i,ii,iii,iv options | -- |
| What should a woman do if she misses 2 pills? | 1. She has to **take 2 pills the next da**y 2. Again 2 pills the second next day. 3. The **couple should also use condom for 7 days** | 1-3 | Any 3/3 of i,ii,iii options | Any 2/3 of i,ii,iii options | Any 1/3 of i,ii,iii options | -- |
| Can OCPs be given to: | - 1. Newly married women? YES/NO   2. Illiterate women? YES/NO   3. Women who do not want any more children? YES/NO | 1-2 for each answer | Yes for all 3/3 of i,ii,iii options | Yes for any 2/3 of i,ii,iii options | Yes for any 1/3 of i,ii,iii options | -- |
| Which OCPs are available in the government supply? | 1. Mala D 2. Mala N | 1-2 or both | Both 2/2 of i,ii options | Any 1/2 of i,ii options | 0/2 of i,ii options | -- |
| What is failure rate of condoms IF USED correctly? | 1. <5% 2. 6-15% 3. >15% 4. Other. Specify___________ 5. Do not know | 1-4 | Option i | N/A | Option ii,iii,iv | -- |
| What kind of contraceptive is DMPA? | i.Medroxyprogesterone acetate. DMPA is a Progestogen-only Injectable (POI) |  | Only option i | N/A | Other than option i | -- |
| If a woman wishes to use DMPA, what questions do you need to ask her in history? | 1. Pregnancy 2. Irregular periods 3. Breast cancer 4. Liver disease 5. Thromboembolic episodes (HEART ATTACK/STROKE/TIA) | 1-5 or all or combinations | Any >2/5 of i,ii,iii,iv,v options | Any 2/5 of i,ii,iii,iv,v options | Any 0-1/5 of i,ii,iii,iv,v options | -- |
| If a woman wishes to use DMPA, what are the most important issues on which you should counsel her? | 1. Menstruation related side effects 2. Delayed return of fertility 3. Don’t know | 1-2 or both | Both 2/2 of option i,ii | Any 1/2 option i,ii | 0/2 of option i,ii | Option iii |
| Is injectable contraceptive available in public health system? | 1. Yes 2. No | 1 or 2 | Only option i | N/A | Option ii | -- |
| What are the three prerequisites for lactational amenorrhea to be an effective contraceptive method? | 1. Amenorrhea 2. Exclusive breast feeding 3. 6 months 4. Don’t know | 1-3 or all | Any 2/3 of i,ii,iii options | Any 1/3 of i,ii,iii option | 0/3 of i,ii,iii option | Option iv |
| A woman has delivered a healthy baby 3 months ago. She is breast feeding her baby along with top feed. Which contraceptives can be advised to her? | 1. IUCD 2. Injectable 3. POP 4. Condom | 1-4 or all | Any 3/4 of i,ii,iii,iv options | Any 2/4 of i,ii,iii,iv options | Any 1/4 of i,ii,iii,iv options | -- |
