# Supplementary material for: Competency assessment of the medical interns and nurses and documenting prevailing practices to provide family planning services in teaching hospitals in three states of India
Source: PLoS One. 2019 Nov 6;14(11):e0211168. doi: 10.1371/journal.pone.0211168 (PMC6834278; doi:10.1371/journal.pone.0211168)
Supplement: S1 Table — (DOCX) [file pone.0211168.s005.docx]

**S1 Table: Gender based assessment of knowledge regarding various contraceptive methods in each provider category.**

| **Question assessing knowledge**  ***(expected correct response****)* | **Interns** | | **Nurses** | | **Total** | |  |
| --- | --- | --- | --- | --- | --- | --- | --- |
|  | **Male**  **N=41**  **(50.6%)** | **Female**  **N=40**  **(49.4%)** | **Male**  **N=13**  **(15.9%)** | **Female**  **N=69**  **(84.1%)** | **Male**  **N=54 (33.1%)** | **Female**  **N=109 (66.9%)** | **Chi-square (p-value)** |
| 1. What are the various family planning methods you know of? |  |  |  |  |  |  |  |
| - Condom | 41(100) | 40(100) | 12(92.3) | 62(89.9) | 53(98.1) | 102(93.6) | 1.61 (0.204) |
| - IUCD | 38(92.7) | 38(95) | 11(84.6) | 66(95.7) | 49(90.7) | 104(95.4) | 1.36 (0.242) |
| - OCP | 39(95.1) | 40(100) | 11(84.6) | 66(95.7) | 50(92.6) | 106(97.2) | 1.90 (0.168) |
| - Emergency Contraceptive Pill | 15(36.6) | 20(50) | 5(38.5) | 14(20.3) | 20(37.0) | 34(31.2) | 0.55 (0.456) |
| - Injectable Contraceptive | 14(34.1) | 20(50) | 3(23.1) | 31(44.9) | 17(31.5) | 51(46.8) | 3.48 (0.062) |
| - Natural Method | 24(58.5) | 29(72.5) | 5(38.5) | 40(58) | 29(53.7) | 69(63.3) | 1.38 (0.239) |
| - Implantable Contraceptive | 6(14.6) | 9(22.5) | 2(15.4) | 7(10.1) | 8(14.8) | 16(14.7) | 0.00 (0.982) |
| - Non-hormonal non-steroidal pill | 1(2.4) | 3(7.5) | 2(15.4) | 4(5.8) | 3(5.6) | 7(6.4) | 0.04 (0.828) |
| - Permanent Contraception | 35(85.4) | 36(90) | 11(84.6) | 58(84.1) | 46(85.2) | 94(86.2) | 0.03 (0.856) |
| - Spermicides | 5(12.2) | 8(20) | **2(15.4)*** | **1(1.4)*** | 7(13.0) | 9(8.3) | 0.90 (0.342) |
| 1. Choice of Contraceptive for newly married couple? *(at least 2 options out of Condoms/OCP/POP/IUCD)* | 9(22) | 14(35) | 5(38.5) | 18(26.1) | 14 (25.9) | 32(29.4) | 4.94 (0.084) |
| 1. Choice of Contraceptive for a woman with one child. *(at least 2 options out of Condoms/OCP/POP/IUCD)* | 13(31.7) | 13(32.5) | 5(38.5) | 30(43.5) | 18(33.3) | 43(39.4) | 3.93 (0.140) |
| 1. Choice of Contraceptive for women with three children *(at least 2 options out of Condoms/OCP/POP/IUCD, sterilization)* | **12(30)*** | **10(25)*** | 5(38.5) | 21(30.4) | 17(32.1) | 31(28.4) | **6.23 (0.044)** |
| 1. Contraceptives can be given to a newly married 20 years old women coming alone to your clinic | 0 | 0 | 8(61.5) | 29(42) | 8 (14.8) | 29(26.6) | **10.28 (0.006)** |
| 1. Contraceptives can be given to an unmarried woman coming alone to your clinic | 0 | 0 | 6(46.2) | 26(37.7) | 6 (11.1) | 26 (23.9) | **10.83 (0.013)** |
| 1. It is legal in India to provide contraceptives to unmarried people | 0 | 0 | 5(38.5) | 28(40.6) | 5 (9.3) | 28(25.7) | **12.42 (0.006)** |
| 1. How many types of IUDs are you aware of?   *(any 2 options out of Copper/Hormonal/First generation/ Inert IUCD)* | **27(65.9)*** | **36(90)*** | 3(27.3) | 23(34.3) | 30 (57.7) | 59(55.1) | 0.09 (0.761) |
| 1. What are the three common conditions you will rule out before inserting CuT? *(any 3 options out of Pregnancy, STI/HIV, Irregular Periods, Adnexal Mass/Ectopic Pregnancy, Multiple Sexual Partners)* | 17(41.5) | 25(62.5) | 5(38.5) | 21(30.4) | 22(40.7) | 46(42.2) | 0.79 (0.850) |
| 1. What are the most common side effects of CuT insertion? *(any 2 options out of Pain/cramps, Bleeding /menorrhagia/ spotting/irregular bleeding, Infections/PID/vaginal discharge, Expulsions)* | 16(39) | 25(62.5) | 5(38.5) | 24(34.8) | 21(38.9) | 49(45.0) | 1.41 (0.493) |
| 1. What type of CuT is available in Govt supply? *(CuT 375/ CuT 380A)* | 14(34.1) | 10(25) | 3(23.1) | 16(23.2) | 17(31.5) | 26(23.9) | 4.12 (0.249) |
| 1. How long CuT 380A provide protection for? *(10 years)* | 18(43.9) | 24(60) | 4(30.8) | 25(36.2) | 22(40.7) | 49(45.0) | 0.73 (0.693) |
| 1. When is Post-Partum IUCD to be inserted?   *[Within 10 minutes of delivery (early), Within 48 hours of delivery (late), During Caesarean section]* | 2(4.9) | 2(5) | 0(0) | 2(2.9) | 2(3.7) | 4(3.7) | 0.20 (0.976) |
| 1. When should consent be taken for PPIUCD?   *(at least 2 options out of these: antenatal period, early labour, early post-natal period with in 48 hours of delivery; before Caesarean section, to be considered as correct).* | 3(7.3) | 7(17.5) | 0(0) | 9(13) | **3(5.6)*** | **16(14.7)*** | **8.31 (0.040)** |
| 1. Conditions to rule out before prescribing OCPs?  *(at least 4 options out of these: H/o Smoking, Diabetes, Headaches, Cardiovascular diseases, Thromboembolic episodes, Less than 6 weeks postpartum, Liver disease, Breast cancer)* | 5(12.2) | 13(32.5) | 1(7.7) | 10(14.5) | 6(11.1) | 23(21.1) | 3.38 (0.336) |
| 1. OCPs can be bought over the counter | 31(75.6) | 22(55) | 9(69.2) | 40(58) | 40 (74.1) | 62(56.9) | 5.27 (0.072) |
| 1. Instruction to be given to a woman who wants to use OCPs? *(at least 3 options out of these: When to start the pill, Daily intake without fail (3 weeks + 1week), What to do if she misses a pill, Side effects)* | **21(51.2)*** | **31(77.5)*** | 4(30.8) | 32(46.4) | 25(46.3) | 63(57.8) | 6.16 (0.104) |
| 1. What should a woman do if she misses two pills?   *(all three options: she has to take 2 pills the next day, Again 2 pills the second next day, The couple should also use condom for 7 days)* | 4(9.8) | 5(12.5) | 0(0) | 3(4.3) | 4(7.4) | 8(7.3) | 0.66 (0.881) |
| 1. OCPs can be given to a newly married woman | 34(82.9) | 34(85) | 8(61.5) | 35(50.7) | 42(77.8) | 69(63.3) | 3.48 (0.062) |
| 1. OCPs can be given to an illiterate woman | 32(78) | 32(80) | 9(69.2) | 51(73.9) | 41(75.9) | 83(76.1) | 0.00 (0.975) |
| 1. OCPs can be given to a woman who do not want any more children | 24(58.5) | 29(72.5) | 12(92.3) | 49(71) | 36(66.7) | 78(71.6) | 0.41 (0.521) |
| 1. Which OCP is available in Govt Supply? *(MALA N)* | 39(95.1) | 38(95) | 10(76.9) | 52(75.4) | 49(90.7) | 90(82.6) | 3.42 (0.180) |
| 1. What is the failure rate of condom if used correctly? *(<5%)* | 16(39) | 18(45) | 3(23.1) | 14(20.3) | **19(35.2)*** | **32(29.4)*** | **8.29 (0.016)** |
| 1. What are the two most common advantages of using a Condom? (*Minimum side effects, protection from STI/HIV)* | 7(17.1) | 10(25) | 2(15.4) | 17(24.6) | **9(16.7)*** | **27(24.8)*** | **7.80 (0.050)** |
| 1. What kind of Contraceptive is DMPA? *[Depot Medroxyprogesterone acetate. DMPA is a Progestogen-only Injectable (POI)]* | 25(61) | 34(85) | 3(23.1) | 29(42) | 28(51.9) | 63(57.8) | 2.50 (0.285) |
| 1. What questions to ask a woman in history before prescribing DMPA? *[at least 2 out of these options: Pregnancy, Irregular periods, Breast cancer, Liver disease, Thromboembolic episodes (Heart attack /Stroke/TIA)]* | 5(12.2) | 8(20) | 1(7.7) | 10(14.5) | 6(11.1) | 18(16.5) | 1.24 (0.742) |
| 1. If a woman wishes to use DMPA, what are the most important issues on which you should counsel her? *(Menstruation related side effects, Delayed return of fertility)* | 2(4.9) | 8(20) | 1(7.7) | 4(5.8) | 3(5.6) | 12(11.0) | 1.36 (0.505) |
| 1. Injectable contraceptives are available in government supply | 16(39) | 20(50) | 2(15.4) | 25(36.2) | 18(33.3) | 45(41.3) | 1.30 (0.522) |
| 1. What are the three prerequisites for lactational amenorrhea to be an effective contraceptive method?   *(all three options: Amenorrhea, Exclusive breast feeding, duration of 6 months)* | 19(46.3) | 21(52.5) | 6(46.2) | 29(42) | 25(46.3) | 50(45.9) | 0.15 (0.926) |
| 1. A woman has delivered a healthy baby 3 months ago. She is breast feeding her baby along with top feed. Which contraceptives can be advised to her? *(atleast 3 out of these options: IUCD, Injectable, POP ,Condom)* | 7(17.1) | 5(12.5) | 0(0) | 11(15.9) | 7(13.0) | 16(14.7) | 2.31 (0.510) |
| 1. What is the type of contraception used after unprotected intercourse? *(Emergency contraception)* | 10(24.4) | 19(47.5) | 1(7.7) | 4(5.8) | **11(20.4)*** | **23(21.1)*** | **17.75 (0.001)** |
| 1. Till what time emergency contraceptive pill is effective? *(within 72 hours)* | 39(95.1) | 39(97.5) | 10(76.9) | 59(85.5) | 49(90.7) | 98(89.9) | 0.78 (0.675) |
| 1. How frequently should centchroman be taken?   *(twice weekly for first 3 months and then weekly)* | 23(56.1) | 21(52.5) | **5(38.5)*** | **21(30.4)*** | 10(18.5) | 10(9.2) | 4.84 (0.089) |

**p<0.05*
