# Supplementary material for: Competency assessment of the medical interns and nurses and documenting prevailing practices to provide family planning services in teaching hospitals in three states of India
Source: PLoS One. 2019 Nov 6;14(11):e0211168. doi: 10.1371/journal.pone.0211168 (PMC6834278; doi:10.1371/journal.pone.0211168)
Supplement: S2 Table — (DOCX) [file pone.0211168.s006.docx]

**S2 Table: Age wise assessment of knowledge regarding various contraceptive methods of the study participants.**

| **Question assessing knowledge**  ***(expected correct response****)* | **Total** | | **Chi-square (p-value)** |
| --- | --- | --- | --- |
|  | **< 25 years** | **>25 years** |  |
| Total | 84 (100) | 79 (100) |  |
| 1. What are the various family planning methods you know of? |  |  |  |
| - Condom | 83(98.8) | 72(91.1) | **5.13(0.023)** |
| - IUCD | 79(94.0) | 74(93.7) | 0.01(0.920) |
| - OCP | 81(96.4) | 75(94.9) | 0.22(0.639) |
| - Emergency Contraceptive Pill | 39(46.4) | 15(19.0) | **13.83(0.000)** |
| - Injectable Contraceptive | 35(41.7) | 33(41.8) | 0.00(0.989) |
| - Natural Method | 54(64.3) | 44(55.7) | 1.25(0.263) |
| - Implantable Contraceptive | 15(17.9) | 9(11.4) | 1.35(0.244) |
| - Non-hormonal non-steroidal pill | 4(4.8) | 6(7.6) | 0.56(0.451) |
| - Permanent Contraception | 73(86.9) | 67(84.8) | 0.14(0.701) |
| - Spermicides | 13(15.5) | 3(3.8) | **6.27(0.012)** |
| 1. Choice of Contraceptive for newly married couple? *(at least 2 options out of Condoms/OCP/POP/IUCD)* | 26(31.0) | 20(25.3) | **11.28(0.004)** |
| 1. Choice of Contraceptive for a woman with one child. *(at least 2 options out of Condoms/OCP/POP/IUCD)* | 29(34.5) | 32(40.5) | 0.79(0.673) |
| 1. Choice of Contraceptive for women with three children *(at least 2 options out of Condoms/OCP/POP/IUCD, sterilization)* | 22(26.5) | 26(32.9) | 1.84(0.399) |
| 1. Contraceptives can be given to a newly married 20 years old women coming alone to your clinic | 5(6.0) | **32(40.5)** | **86.08(0.000)** |
| 1. Contraceptives can be given to an unmarried woman coming alone to your clinic | 2(2.4) | **30(38.0)** | **76.52(0.000)** |
| 1. It is legal in India to provide contraceptives to unmarried people | 3(3.6) | **30(38.0)** | **63.15(0.000)** |
| 1. How many types of IUDs are you aware of?   *(any 2 options out of Copper/Hormonal/First generation/ Inert IUCD)* | 61(73.5) | **28(36.8)** | **21.62(0.000)** |
| 1. What are the three common conditions you will rule out before inserting CuT? *(any 3 options out of Pregnancy, STI/HIV, Irregular Periods, Adnexal Mass/Ectopic Pregnancy, Multiple Sexual Partners)* | 44(52.4) | 24(30.4) | **10.30(0.016)** |
| 1. What are the most common side effects of CuT insertion? *(any 2 options out of Pain/cramps, Bleeding/menorrhagia/spotting/irregular bleeding, Infections/PID/vaginal discharge, Expulsions)* | 41(48.8) | 29(36.7) | 2.45(0.293) |
| 1. What type of CuT is available in Govt supply? *(CuT 375/ CuT 380A)* | 23(27.4) | 20(25.3) | 6.94(0.074) |
| 1. How long CuT 380A provide protection for? *(10 years)* | 41(48.8) | 30(38.0) | 3.90(0.142) |
| 1. When is Post-Partum IUCD to be inserted?  *[at least 2 options out of these: Within 10 minutes of delivery (early), Within 48 hours of delivery (late), During Caesarean section]* | 5(6.0) | 1(1.3) | **10.42(0.015)** |
| 1. When should consent be taken for PPIUCD?   *(at least 2 options out of these: antenatal period, early labour, early post-natal period with in 48 hours of delivery; before Caesarean section, to be considered as correct).* | 10(11.9) | 9(11.4) | 3.01(0.389) |
| 1. Conditions to rule out before prescribing OCPs?  *(at least 4 options out of these: H/o Smoking, Diabetes, Headaches, Cardiovascular diseases, Thromboembolic episodes, Less than 6 weeks postpartum, Liver disease, Breast cancer)* | 17(20.2) | 12(15.2) | **16.08(0.001)** |
| 1. OCPs can be bought over the counter | 56(66.7) | 46(58.2) | 3.77(0.152) |
| 1. Instruction to be given to a woman who wants to use OCPs? *(at least 3 options out of these: When to start the pill, Daily intake without fail (3 weeks + 1week), What to do if she misses a pill, Side effects)* | 53(63.1) | 35(44.3) | **11.20(0.011)** |
| 1. What should a woman do if she misses two pills?   *(all three options: she has to take 2 pills the next day, Again 2 pills the second next day, The couple should also use condom for 7 days)* | 10(11.9) | 2(2.5) | **8.25(0.041)** |
| 1. OCPs can be given to a newly married woman | 70(83.3) | 41(51.9) | **18.51(0.000)** |
| 1. OCPs can be given to an illiterate woman | 65(77.4) | 59(74.7) | 0.16(0.687) |
| 1. OCPs can be given to a woman who do not want any more children | 57(67.9) | 57(72.2) | 0.35(0.550) |
| 1. Which OCP is available in Govt Supply? *(MALA N)* | 78(92.9) | 61(77.2) | **10.93(0.004)** |
| 1. What is the failure rate of condom if used correctly? *(<5%)* | 32(38.1) | 19(24.1) | **15.64(0.000)** |
| 1. What are the two most common advantages of using a Condom? (*Minimum side effects, protection from STI/HIV)* | 20(23.8) | 16(20.3) | **7.79(0.050)** |
| 1. What kind of Contraceptive is DMPA? *[Depot Medroxyprogesterone acetate. DMPA is a Progestogen-only Injectable (POI)]* | 57(67.9) | 34(43.0) | **11.68(0.003)** |
| 1. What questions to ask a woman in history before prescribing DMPA? *[at least 2 out of these options: Pregnancy, Irregular periods, Breast cancer, Liver disease, Thromboembolic episodes (Heart attack /Stroke/TIA)]* | 14(16.7) | 10(12.7) | 6.40(0.093) |
| 1. If a woman wishes to use DMPA, what are the most important issues on which you should counsel her? *(Menstruation related side effects, Delayed return of fertility)* | 9(10.7) | 6(7.6) | 4.32(0.115) |
| 1. Injectable contraceptives are available in government supply | 34(40.5) | 29(36.7) | 5.43(0.066) |
| 1. What are the three prerequisites for lactational amenorrhea to be an effective contraceptive method? *(all three options: Amenorrhea, Exclusive breast feeding, duration of 6 months)* | 43(51.2) | 32(40.5) | **6.17(0.046)** |
| 1. A woman has delivered a healthy baby 3 months ago. She is breast feeding her baby along with top feed. Which contraceptives can be advised to her? *(at least 3 out of these options: IUCD, Injectable, POP ,Condom)* | 14(16.7) | 9(11.4) | 2.11(0.549) |
| 1. What is the type of contraception used after unprotected intercourse? *(Emergency contraception)* | 26(31.0) | 8(10.1) | **47.77(0.000)** |
| 1. Till what time emergency contraceptive pill is effective? *(within 72 hours)* | 79(94.0) | 68(86.1) | 2.95(0.228) |
| 1. How frequently should centchroman be taken?   *(twice weekly for first 3 months and then weekly)* | 18(21.4) | 2(2.5) | **21.19(0.000)** |
| 1. A woman has used Emergency contraceptive pills 3 times in last 1 year. Now she comes to your OPD and ask for it the fourth time. Will you prescribe it again this time? | 44(52.4) | 26(32.9) | **6.67(0.036)** |
| 1. How is centchroman (Chhaya/Saheli) different from OCPs? | 21(25.0) | 5(6.3) | **21.74(0.000)** |
