# Supplementary material for: Competency assessment of the medical interns and nurses and documenting prevailing practices to provide family planning services in teaching hospitals in three states of India
Source: PLoS One. 2019 Nov 6;14(11):e0211168. doi: 10.1371/journal.pone.0211168 (PMC6834278; doi:10.1371/journal.pone.0211168)
Supplement: S3 Table — (DOCX) [file pone.0211168.s007.docx]

**S3 Table: Assessment of knowledge regarding various contraceptive methods as per the marital status of the study participants.**

| **Question assessing knowledge**  ***(expected correct response****)* | **Interns** | | **Nurses** | | **Total** | | |
| --- | --- | --- | --- | --- | --- | --- | --- |
|  | **Married** | **Un-married** | **Married** | **Un-married** | **Married** | **Un-married** | **Chi-square (p-value)** |
|  | **N=7**  **(8.6%)** | **N=74 (91.4%)** | **N= 69(85.4%)** | **N=12**  **(14.2%)** | **N=77**  **(47.2%)** | **N=86**  **(52.8%)** |  |
| 1. What are the various family planning methods you know of? |  |  |  |  |  |  |  |
| - Condom | 7(100) | 74(100) | 64(91.4) | 10(83.3) | 71(92.2) | 84(97.7) | 2.60(0.107) |
| - IUCD | 7(100) | 69(93.2) | 66(94.3) | 11(91.7) | 73(94.8) | 80(93.0) | 0.22(0.636) |
| - OCP | **6(85.7)*** | **73(98.6)*** | 66(94.3) | 11(91.7) | 72(93.5) | 84(97.7) | 1.71(0.190) |
| - Emergency Contraceptive Pill | **0** | **35(47.3)*** | 14(20.0) | 5(41.7) | 14(18.2) | 40(46.5) | **14.71(0.000)** |
| - Injectable Contraceptive | 3(42.9) | 31(41.9) | 29(41.4) | 5(41.7) | 32(41.6) | 36(41.9) | 0.00(0.969) |
| - Natural Method | 5(71.4) | 48(64.9) | 37(52.9) | 8(66.7) | 42(54.5) | 56(65.1) | 1.89(0.169) |
| - Implantable Contraceptive | 1(14.3) | 14(18.9) | 9(13) | 0(0) | 10(13.0) | 14(16.3) | 0.35(0.554) |
| - Non-hormonal non-steroidal pill | 0(0) | 4(5.4) | 6(8.7) | 0(0) | 6(7.8) | 4(4.7) | 0.69(0.404) |
| - Permanent Contraception | 7(100) | 64(86.5) | 58(84.1) | 11(91.7) | 65(84.4) | 75(87.2) | 0.26(0.609) |
| - Spermicides | 2(28.6) | 11(14.9) | 2(2.9) | 1(8.3) | 4(5.2) | 12(14.0) | 3.52(0.061) |
| 1. Choice of Contraceptive for newly married couple? *(at least 2 options out of Condoms/OCP/POP/IUCD)* | 3(42.9) | 20(27) | 22(31.9) | 1(8.3) | 25(32.5) | 21(24.4) | **9.22(0.010)** |
| 1. Choice of Contraceptive for a woman with one child. *(at least 2 options out of Condoms/OCP/POP/IUCD)* | 1(14.3) | 25(33.8) | 32(46.4) | 3(25) | 33(42.9) | 28(32.6) | 2.16(0.339) |
| 1. Choice of Contraceptive for women with three children *(at least 2 options out of Condoms/OCP/POP/IUCD, sterilization)* | 0 | 22(30.1) | 22(31.9) | 4(33.3) | 22(28.6) | 26(30.6) | 0.10(0.951) |
| 1. Contraceptives can be given to a newly married 20 years old women coming alone to your clinic | 0 | 0 | 31(44.9) | 5(41.7) | 32(41.6) | 5(5.8) | **72.44(0.000)** |
| 1. Contraceptives can be given to an unmarried woman coming alone to your clinic | 0 | 0 | 26(37.7) | 5(41.7) | 27(35.1) | 5(5.8) | **63.52(0.000)** |
| 1. It is legal in India to provide contraceptives to unmarried people | 0 | 0 | 29(42) | 3(25) | 30(39.0) | 3(3.5) | **57.77(0.000)** |
| 1. How many types of IUDs are you aware of?   *(any 2 options out of Copper/Hormonal/First generation/ Inert IUCD)* | 5(71.4) | 58(78.4) | 21(32.3) | 5(41.7) | 26(35.6) | 63(73.3) | **22.69(0.000)** |
| 1. What are the three common conditions you will rule out before inserting CuT? *(any 3 options out of Pregnancy, STI/HIV, Irregular Periods, Adnexal Mass/Ectopic Pregnancy, Multiple Sexual Partners)* | 2(28.6) | 40(54.1) | 22(31.9) | 3(25) | 25(32.5) | 43(50.0) | 6.43(0.092) |
| 1. What are the most common side effects of CuT insertion? *(any 2 options out of Pain/cramps, Bleeding/menorrhagia/spotting/irregular bleeding, Infections/PID/vaginal discharge, Expulsions)* | 2(28.6) | 39(52.7) | 23(33.3) | 5(41.7) | 26(33.8) | 44(51.2) | 5.79(0.055) |
| 1. What type of CuT is available in Govt supply? *(CuT 375/ CuT 380A)* | 4(57.1) | 20(27) | 17(24.6) | 2(16.7) | **21(27.3)** | 22(25.6) | **11.76(0.008)** |
| 1. How long CuT 380A provide protection for? *(10 years)* | 3(42.9) | 39(52.7) | **25(36.2)*** | **3(25)*** | 29(37.7) | 42(48.8) | 3.01(0.222) |
| 1. *When is Post-Partum IUCD to be inserted?*   *(at least 2 options out of these: [Within 10 minutes of delivery (early), Within 48 hours of delivery (late), During Caesarean section]* | 1(14.3) | 3(4.1) | 2(2.9) | 0(0) | 3(3.9) | 3(3.5) | **8.43(0.038)** |
| 1. When should consent be taken for PPIUCD?   *at least 2 options out of these: (at least 2 options out of these: antenatal period, early labour, early post-natal period with in 48 hours of delivery; before Caesarean section, to be considered as correct).* | 0(0) | 10(13.5) | 8(11.6) | 1(8.3) | 8(10.4) | 11(12.8) | 1.39(0.706) |
| 1. Conditions to rule out before prescribing OCPs? *(at least 4 options out of these: H/o Smoking, Diabetes, Headaches, Cardiovascular diseases, Thromboembolic episodes, Less than 6 weeks postpartum, Liver disease, Breast cancer)* | 1(14.3) | 17(23) | 11(15.9) | 0(0) | 12(15.6) | 17(19.8) | **14.59(0.002)** |
| 1. OCPs can be bought over the counter | 5(71.4) | 48(64.9) | 43(62.3) | 5(41.7) | 49(63.6) | 53(61.6) | 0.14(0.930) |
| 1. Instruction to be given to a woman who wants to use OCPs? *(at least 3 options out of these: When to start the pill, Daily intake without fail (3 weeks + 1week), What to do if she misses a pill, Side effects)* | 6(85.7) | 46(62.2) | 31(44.9) | 5(41.7) | 37(48.1) | 51(59.3) | 5.38(0.146) |
| 1. *What should a woman do if she misses two pills?*   *(all three options: she has to take 2 pills the next day, Again 2 pills the second next day, The couple should also use condom for 7 days)* | 0(0) | 9(12.2) | 3(4.3) | 0(0) | 3(3.9) | 9(10.5) | 4.38(0.223) |
| 1. OCPs can be given to a newly married woman | 6(85.7) | 62(83.8) | 34(49.3) | 8(66.7) | 41(53.2) | **70(81.4)** | **14.81(0.000)** |
| 1. OCPs can be given to an illiterate woman | 5(71.4) | 59(79.7) | 49(71) | 10(83.3) | 55(71.4) | 69(80.2) | 1.730(0.188) |
| 1. OCPs can be given to a woman who do not want any more children | 5(71.4) | 48(64.9) | 50(72.5) | 10(83.3) | 56(72.7) | 58(67.4) | 0.540(0.463) |
| 1. Which OCP is available in Govt Supply? *(MALA N)* | 7(100) | 70(94.6) | 53(76.8) | 8(66.7) | 61(79.2) | 78(90.7) | 5.599(0.061) |
| 1. What is the failure rate of condom if used correctly? *(<5%)* | 4(57.1) | 30(40.5) | 14(20.3) | 3(25) | 18(23.4) | 33(38.4) | **7.922(0.019)** |
| 1. What are the two most common advantages of using a Condom? (*Minimum side effects, protection from STI/HIV)* | 3(42.9) | 14(18.9) | 14(20.3) | 5(41.7) | 17(22.1) | 19(22.1) | 5.676(0.128) |
| 1. What kind of Contraceptive is DMPA? *[Depot Medroxyprogesterone acetate. DMPA is a Progestogen-only Injectable (POI)]* | **3(42.9)*** | **56(75.7)*** | 27(39.1) | 4(33.3) | 31(40.3) | 60(69.8) | **14.799(0.001)** |
| 1. What questions to ask a woman in history before prescribing DMPA? *[at least 2 out of these options: Pregnancy, Irregular periods, Breast cancer, Liver disease, Thromboembolic episodes (Heart attack /Stroke/TIA)]* | 0(0) | 13(17.6) | 8(11.6) | 2(16.7) | 9(11.7) | 15(17.4) | 2.931(0.402) |
| 1. If a woman wishes to use DMPA, what are the most important issues on which you should counsel her? *(Menstruation related side effects, Delayed return of fertility)* | 0(0) | 10(13.5) | 5(7.2) | 0(0) | 5(6.5) | 10(11.6) | 2.411(0.300) |
| 1. Injectable contraceptives are available in government supply | 5(71.4) | 31(41.9) | 23(33.3) | 3(25) | 29(37.7) | 34(39.5) | 1.867(0.393) |
| 1. What are the three prerequisites for lactational amenorrhea to be an effective contraceptive method? *(all three options: Amenorrhea, Exclusive breast feeding, duration of 6 months)* | 3(42.9) | 37(50) | 32(46.4) | 3(25) | 35(45.5) | 40(46.5) | 2.431(0.297) |
| 1. A woman has delivered a healthy baby 3 months ago. She is breast feeding her baby along with top feed. Which contraceptives can be advised to her? *(at least 3 out of these options: IUCD, Injectable, POP, Condom)* | 1(14.3) | 11(14.9) | **10(14.5)*** | **1(8.3)*** | 11(14.3) | 12(14.0) | 1.187(0.756) |
| 1. What is the type of contraception used after unprotected intercourse? *(Emergency contraception)* | 3(42.9) | 26(35.1) | 4(5.8) | 1(8.3) | 7(9.1) | **27(31.4)** | **28.416(0.000)** |
| 1. Till what time emergency contraceptive pill is effective? *(within 72 hours)* | 7(100) | 71(95.9) | 60(87) | 9(75) | 67(87.0) | 80(93.0) | 2.056(0.358) |
| 1. How frequently should centchroman be taken?  *(twice weekly for first 3 months and then weekly)* | 3(42.9) | 41(55.4) | 23(33.3) | 2(16.7) | 2(2.6) | **18(20.9)** | **22.971(0.000)** |
| 1. A woman has used Emergency contraceptive pills 3 times in last 1 year. Now she comes to your OPD and ask for it the fourth time. Will you prescribe it again this time? | 1(14.3) | 22(29.7) | 3(4.3) | 0(0) | 27(35.1) | **43(50.0)** | **7.628(0.022)** |
| 1. How is centchroman (Chhaya/Saheli) different from OCPs? | 0(0) | 18(24.3) | 2(2.9) | 0(0) | 4(5.2) | **22(25.6)** | **20.082(0.000)** |

**p<0.0;* bold figures, depicts statistically significant difference
