# Supplementary material for: Competency assessment of the medical interns and nurses and documenting prevailing practices to provide family planning services in teaching hospitals in three states of India
Source: PLoS One. 2019 Nov 6;14(11):e0211168. doi: 10.1371/journal.pone.0211168 (PMC6834278; doi:10.1371/journal.pone.0211168)
Supplement: S4 Table — (DOCX) [file pone.0211168.s008.docx]

**S4 Table: Teaching practices of faculty of Community Medicine and Gynaecology and Obstetrics in the medical colleges related to family planning services.**

| **State*** | **New Delhi** | **Rajasthan** | | **Maharashtra** | | | **Total**  **N=6** |
| --- | --- | --- | --- | --- | --- | --- | --- |
| **Medical College (MC)** | **MC1** | **MC2** | **MC3** | **MC4** | **MC5** | **MC6** |  |
| **Department of Faculty interviewed** | Community Medicine | Community Medicine | Community Medicine | Obstetrics and Gynecology | Obstetrics and Gynecology | Obstetrics and Gynecology |  |
| **Where do students receive family planning practical training?** |  |  |  |  |  |  |  |
| - Family planning Room | **√** | - | **√** | **√** | **√** | - | 4/6 |
| - Obstetrics and Gynaecology OPD | **√** | **√** | - | **√** | - | **√** | 4/6 |
| **What facilities are available to train students on FP?** |  |  |  |  |  |  |  |
| - Skill Lab | **-** | **-** | - | **√** | **√** | - | 2/6 |
| - Dummy | **√** | **√** | - | - | - | - | 2/6 |
| - Model | - | **√** | - | - | - | - | 1/6 |
| - Patients | - | **√** | **√** | **√** | - | **√** | 4/6 |
| **Samples of contraceptives available for demo** |  |  |  |  |  |  |  |
| - Cu IUCD | **√** | **√** | **√** | **√** | **√** | **√** | 6/6 |
| - Hormonal IUCD | **√** | - | **√** | **√** | **√** | **√** | 5/6 |
| - Combined oral Pill | **√** | **√** | **√** | **√** | **√** | **√** | 6/6 |
| - Progesterone only pill | **√** | - | **√** | **√** | **√** | **√** | 5/6 |
| - DMPA | **√** | **√** | **√** | **√** | **√** | **√** | 6/6 |
| - Condoms | **√** | **√** | **√** | **√** | **√** | **√** | 6/6 |
| - Implants | **√** | - | **√** | **√** | **√** | **√** | 5/6 |
| - Spermicides | **√** | - | **√** | - | **√** | - | 3/6 |
| - Emergency Contraceptive Pill | **√** | **√** | **√** | **√** | **√** | **√** | 6/6 |
| **What training modules are available for FP practical training?** |  |  |  |  |  |  |  |
| - Text Books | **√** | **√** | **√** | **√** | **√** | **√** | 6/6 |
| - GOI Guidelines | - | **√** | **√** | - | - | - | 2/6 |
| **Is Medical eligibility checklist wheel available?** | - | **√** | - | - | - | - | 1/6 |
| **Frequency of students visits in the clinics** | Weekly | Weekly | Weekly | Weekly | Weekly | Weekly | 6/6 |
| **Do you allow them to practice hands on?** | **√** | **√** | **√** | **√** | **√** | **√** | 6/6 |

*OPD: Out-patient department; IUCD: Intrauterine contraceptive device; DMPA: Depot medroxy progesterone acetate; GOI: Government of India Guidelines
