# Supplementary material for: Competency assessment of the medical interns and nurses and documenting prevailing practices to provide family planning services in teaching hospitals in three states of India
Source: PLoS One. 2019 Nov 6;14(11):e0211168. doi: 10.1371/journal.pone.0211168 (PMC6834278; doi:10.1371/journal.pone.0211168)
Supplement: S5 Table — (DOCX) [file pone.0211168.s009.docx]

**S5 Table: Record review and observation of family planning clinics under department of obstetrics and gynaecology or community medicine in the medical colleges.**

| **State^∉^** | **Rajasthan** | | | **Maharashtra** | | | **Total Health Facilities**  **N=6** |
| --- | --- | --- | --- | --- | --- | --- | --- |
| **Medical College (MC)^** | **MC2*** | | **MC3** | **MC4** | **MC5** | **MC6** |  |
| **Department**  **(Health Facility)** | Obs/Gynae  (HF1) | Obs/Gynae  (HF2) | CHC, Community Medicine  (HF3) | Obs/Gynae  (HF3) | Obs/Gynae  (HF4) | Obs/Gynae  (HF) |  |
| 1. **Counselling of clients** |  |  |  |  |  |  |  |
| - Privacy in OPD | **√** | **√** | **√** | **√** | **√** | **√** | 6/6 |
| - Auditory privacy | Y | Y | Y | N | N | Y | 4/6 |
| 1. **Eligibility of clients** |  |  |  |  |  |  |  |
| - Assessment method | Verbal | Verbal | Verbal | Verbal | Verbal | Verbal | 6/6 |
| - MEC wheel usage | **√** | **-** | **-** | **-** | **-** | **-** | 1/6 |
| - MEC wheel in sufficient number | - | **-** | **-** | **-** | **-** | **-** | 0/6 |
| 1. **No. of persons provided FP services in the last month** |  |  |  |  |  |  | **Availability of facility** |
| - IUCDs(interval) | 30 | 12 | 16 | 6 | 2 | 4 | 6/6 |
| - IUCDs (PPIUD) | 227 | 120 | 9 | 73 | 2 | 20 | 6/6 |
| - Tubectomy | 106 | 27 | 12 | 68 | 57 | 28 | 6/6 |
| - Vasectomyφ | N/A | N/A | N/A | 0 | N/A | 3 | 2/6 |
| - DMPA | 8 | N/A | N/A | 0 | 10 | 8 | 4/6 |
| - OCP | 70 | 11 | 18 | 600 | 3 | 5 | 6/6 |
| - Condoms | 1420 | 6 | 22 | 60 | 5 | 30 | 6/6 |
| - Centchroman | 0 | 0 | 0 | 0 | N/A | 0 | 5/6 |
| - Emergency contraceptives | 0 | 0 | 3 | 0 | 0 | 0 | 6/6 |
| **Availability of contraceptives in the stock in the last month** |  |  |  |  |  |  |  |
| - Oral pills | **√** | **√** | **√** | **√** | **√** | **√** | 6/6 |
| - Condoms | **√** | **√** | **√** | **√** | **√** | **√** | 6/6 |
| - Injectable | **√** | **-** | **-** | **-** | **-** | **√** | 2/6 |
| - Emergency contraceptives | - | - | **√** | **-** | **-** | **√** | 2/6 |
| - Cu T 380 A/375 | **√** | **√** | **√** | **√** | **√** | **√** | 6/6 |
| **Family planning logistics management information system** | **√** | **-** | **-** | **-** | **√** | **√** | 3/6 |

**^∉^**OPD: Out-patient department; IUCD: Intrauterine contraceptive device; DMPA: Depot medroxy progesterone acetate; OCP: Oral contraceptive pill; *MC2 had two attached hospitals with obstetrics and gynaecology departments; # CHC: Community Health Center was attached with community medicine department of MC 3; ^ MC 1: Medical college in Delhi did not permit to visit the family planning clinics under department of obstetrics and gynaecology, hence status was not known; φ NA not available
